# Iron dysregulation in cerebral small vessel disease: A quantitative susceptibility mapping study revealing spatial patterns and cognitive predictive value

**DOI:** 10.1016/j.tjpad.2025.100451

**Published:** 2026-01-01

**Authors:** Pengcheng Liang, Meng Li, Qihao Zhang, Nan Zhang, Yena Che, Yian Gao, Chaofan Sui, Xinyue Zhang, Na Wang, Yuanyuan Wang, Yiwen Chen, Zhenyu Cheng, Changhu Liang, Lingfei Guo, Jing Li

**Affiliations:** a Key Laboratory of Endocrine Glucose & Lipids Metabolism and Brain Aging, Ministry of Education, Department of Radiology, Shandong Provincial Hospital Affiliated to Shandong First Medical University, Jinan, Shandong, China; b Department of Psychiatry and Psychotherapy, Jena University Hospital, Philosophenweg 3, 07743, Jena, Germany; c Department of Radiology, Weill Cornell Medical College, New York. 407 East 61st Street, NY, NY 10065, USA; d Binzhou medical university, Yantai, Shandong, China; e Department of Radiology, Beijing Tsinghua Changgung Hospital, School of Clinical Medicine, Tsinghua Medicine, Tsinghua University, Beijing, China; f China-Japan Friendship Hospital (Institute of Clinical Medical Sciences), Chinese Academy of Medical Sciences & Peking Union Medical College, Beijing, China; g Department of Radiology, Beijing Hospital, National Center of Gerontology, Institute of Geriatric Medicine, Chinese Academy of Medical Sciences & Peking Union Medical College, Beijing, China

**Keywords:** Cerebral small vessel disease, Quantitative susceptibility mapping, White matter hyperintensities, Cognitive impairment, Neuroimaging biomarkers

## Abstract

**Background:**

White matter hyperintensities (WMHs) represent a cardinal feature of cerebral small vessel disease (CSVD), yet iron dysregulation alterations within these lesions and their relationship to cognitive decline remains poorly understood.

**Objectives:**

To characterize iron dysregulation in WMH using quantitative susceptibility mapping (QSM) and examine their relationship with CSVD severity and cognitive function.

**Design:**

Cross-sectional study with longitudinal follow-up component.

**Setting:**

Single-center study at Shandong Provincial Hospital Affiliated with Shandong First Medical University, China.

**Participants:**

299 participants recruited from January 2021 to September 2023, with 71 participants completing longitudinal follow-up (mean interval 20.6 months). Participants were categorized into early CSVD (0 points, *n* = 171), mild CSVD (1 point, *n* = 70), and severe CSVD (≥2 points, *n* = 58) groups based on total burden scoring.

**Intervention:**

None (observational study).

**Measurements:**

3.0T MRI with quantitative susceptibility mapping and diffusion tensor imaging. Spatial analysis examined susceptibility values in WMH cores and perilesional zones (0–2 mm, 2–4 mm, 4–6 mm). Cognitive assessments included Montreal Cognitive Assessment (MoCA), Symbol Digit Modalities Test (SDMT), and other neuropsychological tests.

**Results:**

WMH susceptibility values were significantly lower than normal-appearing white matter (-14.55 vs -7.77 ppb, *P* < 0.001) with progressive increases correlating with CSVD severity (*P* < 0.001). Cross-sectionally, higher WMH susceptibility values correlated with lower MoCA scores (*r* = -0.155, *P* = 0.045). Longitudinally, WMH susceptibility values predicted decline in information processing speed (SDMT: β = -0.247, *P* = 0.042). Spatial analysis revealed distinct patterns with perilesional regions showing intermediate susceptibility values.

**Conclusions:**

Iron dysregulation alterations within WMH provide independent information about cognitive risk in CSVD. QSM emerges as a promising biomarker for monitoring cognitive trajectory and may facilitate early identification of patients at risk for cognitive decline.

## Highlights

• First spatial gradient analysis of iron metabolism in CSVD using quantitative susceptibility mapping.
• WMH susceptibility values predict future decline in information processing speed over 20.6 months.
• Distinct pathophysiological patterns revealed between WMH cores and perilesional regions.
• QSM provides independent cognitive risk information beyond traditional volumetric measures.
• Iron metabolism alterations bridge vascular and neurodegenerative pathways in dementia.

## Graphical abstract

**Figure:**
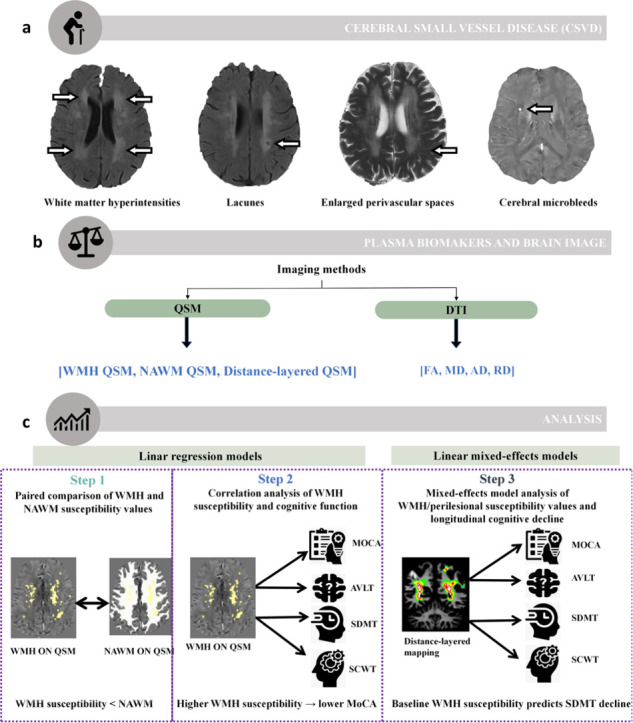

## 1 Introduction

White matter hyperintensity (WMH), a prevalent MRI feature of cerebral small vessel disease (CSVD), is commonly observed in the brains of middle-aged and elderly individuals [1]. Longitudinal studies have demonstrated that the presence of WMH is independently associated with increased risk of adverse outcomes - specifically, individuals with WMH have a threefold increased risk of stroke and a twofold increased risk of dementia. These associations highlight the clinical significance of WMH as both a marker of cerebrovascular dysfunction and a predictor of cognitive decline [2].

Iron is a key nutrient for normal central nervous system development and function. In cerebral white matter injuries, microglia clear iron-rich myelin debris, and the accumulation of myelin debris leads to lipid peroxidation damage in microglia, which is considered a core feature of ferroptosis [3]. Ferroptosis of microglia is also believed to cause neurotoxicity and play a significant role in neurodegenerative diseases. Oligodendrocytes play a critical role in maintaining brain iron homeostasis. Previous studies suggest that glial cells, especially oligodendrocytes, are the most iron-abundant cells in the brain. Moreover, oligodendrocytes are thought to be involved in iron regulation, as they possess proteins responsible for iron storage and transport [4]. Due to the combination of a high metabolic rate with its toxic byproducts, high intracellular iron, and low concentrations of the antioxidative glutathione, oligodendrocytes are particularly vulnerable to oxidative damage. Hence, oxidative damage is a common contributor to oligodendrocyte loss under many pathological conditions like MS and ischemia. Demyelination and oligodendrocyte death is a common feature of inflammatory white matter lesions, both in humans and experimental models [5]. Emerging evidence suggests that dysregulation of iron homeostasis and ferroptosis may play important roles in white matter damage and cognitive decline [3,6,7]. Emerging evidence underscores a potential mechanistic link between demyelination, iron dysregulation, and cognitive impairment, marking a significant stride in understanding CSVD pathophysiology.

Quantitative susceptibility mapping (QSM), an advanced magnetic resonance technique, quantifies local susceptibility changes in tissues influenced by iron content variations and demyelination [[8], [9], [10]]. Previous research has focused on examining the effects of susceptibility values in both the cortex and deep gray matter nuclei, as well as WMH volume, on cognitive functions [11,12]. However, abnormal iron content of WMHs and the relationship between the susceptibility values of WMH and cognitive function have not been established. Current literature shows that QSM provides valuable insights into white matter pathology. Previous studies have demonstrated that simultaneous iron accumulation and demyelination can both lead to increased susceptibility values in white matter lesions [13]. QSM has been established as an effective tool for quantifying biometal content and local susceptibility changes influenced by demyelination [8]. Furthermore, when applied in conjunction with DTI to study white matter pathology, QSM analysis revealed that changes in susceptibility values may reflect both iron loss and myelin degradation [14]. Given the advantages of the QSM in assessing changes in demyelination and iron content, this technology potentially provides a novel perspective for understanding the pathological progression of WMH during CSVD.

Simultaneous iron accumulation and demyelination are indistinguishable from employing QSM alone, given that both lead to signal increases [13]. Diffusion tensor imaging (DTI) enables the non-invasive analysis of pathological processes involved in white matter demyelination. This technique allows us to discern whether susceptibility changes in WMH are due to demyelination, iron accumulation, or a combination of both [15].

Our study aimed to utilize QSM to explore the characteristics of WMH in individuals with CSVD and assess the relationship between WMH susceptibility values and cognitive functions. By comparing WMH susceptibility values with those of normal appearing white matter (NAWM) and analyzing susceptibility value changes across varying CSVD severity levels, we aimed to provide new insights into the pathological changes of WMH in individuals with CSVD and their correlation with cognitive decline. Additionally, our study employed DTI to assess variations in fractional anisotropy (FA), apparent diffusion coefficient (ADC), radial diffusivity (RD), and axial diffusivity (AD) in different stages of CSVD. Demyelination (as myelin is diamagnetic, its loss leads to increased susceptibility values) and iron loss (as iron is paramagnetic, its reduction leads to decreased susceptibility values) have opposing effects on susceptibility values. The observed changes in susceptibility values likely reflect a trade-off between these processes, where the net effect depends on the relative dominance of demyelination or iron loss in WMH. By integrating DTI metrics with susceptibility values, we can better quantify this balance and gain a deeper understanding of the pathological mechanisms underlying WMHs. We also examined the impact of CSVD on the volume of WMH in white matter fiber tracts. This approach will illuminate the progressive damage in white matter fiber tracts, thereby enhancing our understanding of the impact of CSVD at various stages on brain structure. However, previous QSM studies have primarily focused on WMH cores, with limited investigation of perilesional regions that may harbor early pathological changes. Additionally, most existing studies have employed cross-sectional designs, limiting our understanding of the temporal relationship between iron-related changes and cognitive decline. Therefore, the present study aimed to characterize the spatial distribution of susceptibility changes in WMH and surrounding perilesional regions (0–2 mm, 2–4 mm, and 4–6 mm from WMH boundaries), and examine the longitudinal relationship between WMH susceptibility values and cognitive function in CSVD.

## 2 Methods

### 2.1 Participants characteristics

This cross-sectional study was approved by the Institutional Review Board of Shandong Provincial Hospital Affiliated with Shandong First Medical University. All participants signed an informed consent form before the commencement of the study. Participants were recruited from our prospective community-based cohort, the "Neuroimaging Research of Brain Aging in the Elderly in the Community" (ISRCTN13222678), at Shandong Provincial Hospital, through multi-channel recruitment including community-based systematic health screenings, volunteer enrollment, and minority referrals from outpatient clinics (neurology, cardiology, internal medicine). This approach enabled recruitment across the full spectrum of CSVD.

A total of 299 subjects were recruited from January 2021 to September 2023. Inclusion criteria were: (1) age 40–80 years; (2) ability to undergo 3.0T MRI including QSM, DTI, and structural imaging sequences; (3) completion of standardized cognitive assessments; and (4) willingness to participate in potential follow-up evaluations. Importantly, neither cognitive symptoms nor acute cerebrovascular events (TIA/stroke) were required for inclusion, as the study aimed to investigate the entire CSVD spectrum from asymptomatic individuals to those with established disease burden based on neuroimaging findings. For longitudinal analysis, 71 participants completed follow-up evaluations with both imaging and cognitive assessments at a mean interval of 20.6 months. The inclusion criteria for CSVD included manifestations of recent small subcortical infarcts, lacunes (presumed vascular in origin), white matter hyperintensity (presumed vascular in origin), perivascular space, cerebral microbleeds, cortical superficial siderosis, brain atrophy, cortical cerebral microinfarcts, or a score based on the latest MRI consensus criteria for CSVD [16]. The severity of CSVD in individuals was assessed using a scoring system for “total burden of small vessel diseases” [17]. One point indicated the presence of ≥1 lacunar infarct, one point indicated early confluent deep WMH (Fazekas score [18] of 2 or 3) or irregular periventricular WMH extending into deep white matter (Fazekas score of 3), one point indicated moderate to severe (grade 2–3) enlarged perivascular spaces in the basal ganglia, and one point indicated ≥1 cerebral microbleed area. Based on these scores, individuals were categorized into three groups: an early group (0 points), a mild CSVD group (1 point) and a severe CSVD group (2–4 points). The exclusion criteria included the presence of organic brain lesions (such as cerebral apoplexy, brain tumors, and brain trauma), a history of psychiatric or neurological disorders that might influence cognitive functioning, a history of alcohol or substance abuse, acute complications of type 2 diabetes, serious major organ damage (including heart, liver, and kidney), severe hypertension, and severely impaired visual and auditory functions. Two experienced neuroradiologists (LF.G. and J.L.) cross-validated the MRI assessment of CSVD. The two neuroradiologists independently evaluated the images of 100 participants to ensure internal consistency of image interpretation in the present study; the κ coefficients for EPVS (enlarged perivascular spaces) and WMH were 0.73 and 0.81, respectively. Discrepancies in image interpretation were resolved through consultation and discussion with a third higher-level physician.

### 2.2 Clinical and cognitive assessments

In our study, all participants completed the Beijing version of the Montreal Cognitive Assessment (MoCA) (available at www.mocatest.org), a 30-point questionnaire that takes approximately ten minutes to complete [19]. The optimal cutoff scores for cognitive impairment vary according to the educational level of the participants: 13/14 for illiterate individuals, 19/20 for those with 1–6 years of education, and 24/25 for individuals with more than 7 years of education [20]. Participants also completed the Chinese version of the Trail Making Test (TMT), which is divided into two parts: the TMT-A assesses cognitive processing speed, while the TMT-B measures executive function [21]. The sum of the time required for both parts is calculated as the TSUM, reflecting the individual's combined performance in these cognitive tasks. The Auditory Verbal Learning Test (AVLT) assesses memory and learning abilities, typically through auditory verbal recall tasks. The Symbol Digit Modalities Test (SDMT) evaluates information processing speed and visual attention, requiring participants to quickly match symbols with numbers. The Stroop Color and Word Test (SCWT) is used to assess executive function and attention control, particularly in processing information conflicts. The test administrators received specialized training and were blinded to the participants' group assignments.

### 2.3 Laboratory measurements

Venous blood samples were collected under standardized conditions, using EDTA tubes to prevent coagulation. A total of 5 mL of blood was obtained from each participant, followed by centrifugation at 3000 rpm for 15 min to separate plasma. The aliquoted plasma samples were immediately frozen at −20°C for temporary storage and transferred to −70°C for long-term preservation to maintain biomarker stability.

Plasma concentrations of Aβ1–42, total tau (t-Tau), and p-Tau181 were quantified using commercially available enzyme-linked immunosorbent assay (ELISA) kits designed for human proteins (Thermo Fisher/Invitrogen, USA). All assays were conducted in strict accordance with the manufacturer’s instructions to ensure the reliability and reproducibility of the measurements. To minimize inter-assay variability, a single reagent batch was used throughout the analysis process. The optical density of each sample was measured using a microplate reader, and concentrations were calculated based on standard curves generated with reference samples provided in the kits.

### 2.4 Imaging acquisition protocol

In this study, MRI data for all participants were acquired using a 3.0-T MRI scanner (Siemens Healthineers, Erlangen, Germany), equipped with a 32-channel receive head coil. Three-dimensional T1-weighted (3D T1W) structural images were obtained using the Magnetization Prepared Rapid Gradient Echo (MPRAGE) sequence. The parameter settings were as follows: Repetition Time (TR) = 7.3 ms; Echo Time (TE) = 2.4 ms; Inversion Time (TI) = 900 ms; Flip Angle = 9°; Isotropic voxel size = 1 mm^3^, and a 3D multi-echo gradient echo (mGRE) sequence for the QSM (TR = 50 msec, first TE = 6.8 msec, TE interval = 4.1 msec, number of echoes = 10, flip angle = 15, voxel size = 0.9 × 0.9 × 0.9m^3^). Diffusion weighted imaging (DWI) was acquired using a simultaneous multislice (SMS) accelerated single-shot echo planar imaging (EPI) sequence, the settings were as follows: repetition time (TR) = 3000 ms, echo time (TE) = 110 ms, 30 diffusion directions with *b* = 1700 s/mm^2^ and a single *b* = 0 s/mm^2^ acquisition, field of view (FOV) = 220 × 220 mm, matrix size = 110 × 110, 60 slices, and slice thickness = 2.2 mm. Additionally, T2-weighted Fluid Attenuated Inversion Recovery (FLAIR), T2-weighted Turbo Spin Echo were also performed to further identify cerebral abnormalities. T2-Weighted FLAIR Acquisition Parameters: TR/TE/ TI = 5000/387/1800 ms, flip angle = 120°, voxel size = 0.9 × 0.45 × 0.45 mm³, FoV = 230 × 230 mm, slice thickness = 0.9 mm, no interslice gaps. T2-Weighted Scan Parameters: TR/TE = 3200/410 ms, voxel size = 1 × 0.45 × 0.45 mm³, FoV = 230 × 230 mm, slice thickness = 1.0 mm.

### 2.5 Image postprocessing

First of all, structural MRI data were first preprocessed using fMRIPrep 20.1.1 [22]. This preprocessing generated a nonlinear deformation field mapping native space to the MNI2009c space. To obtain the WMH mask, we applied the algorithm developed by Andermatt et al., which employs Multidimensional Gated Recurrent Units with optimizations including data augmentation, selective sampling, residual learning, and DropConnect in the RNN state [23]. This algorithm utilized T1W and FLAIR images to generate the WMH mask in the native T1W space. Before applying the segmentation algorithm, the FLAIR image was rigidly registered to its individual T1-weighted using flirt. With generated WMH masks, we manually corrected inaccuracies in the segmentation for each participant. The algorithm’s parameters were then updated, and segmentation was rerun until accurate segmentation was visually confirmed for all subjects. Additionally, the CAT 12 toolbox (Computational Anatomy Toolbox 12, version 12.8_1977), implemented in Statistical Parametric Mapping (SPM12, r7771, Wellcome Trust Centre for Neuroimaging, University College London), was used to generate GM/WM/CSF masks in native space for each participant. The derived WMH and WM masks facilitated the extraction of the average susceptibility value within WMH and non-WMH WM voxels using fslstats (Figure S1).

In this study, NAWM refers to white matter areas with no visible high signal on FLAIR imaging. This definition is based on visual assessment and segmentation processes. To distinguish NAWM from WMH, we generated “non-WMH masks” through automatic segmentation that excluded areas of signal abnormality. Subsequently, manual correction was performed to further improve the segmentation accuracy. In this way, NAWM represents a region of structurally intact white matter that does not contain any pathological changes visible on imaging.

Brain QSM maps were reconstructed from multi-echo gradient echo (mGRE) complex data using the Morphology Enabled Dipole Inversion with an automatic cerebrospinal fluid (CSF) zero reference algorithm (MEDI+0) [24]. Nonlinear fitting of the multi-echo data was first applied to estimate the total magnetic field, enhancing phase reliability and reducing noise by incorporating information from all available echo times. Subsequently, spatial phase unwrapping was performed to correct for aliasing artifacts in the phase signal, ensuring a smooth and continuous field estimation. Background field contributions, primarily originating from outside the brain, were removed using the Projection onto Dipole Fields (PDF) algorithm, effectively isolating the local magnetic field within the brain [25]. The local field was then inverted into quantitative susceptibility values using the MEDI+0 algorithm, which incorporated structural priors derived from the magnitude image to enforce morphological consistency. A regularization term was included to ensure uniform susceptibility distribution within the CSF, further enhancing reconstruction quality. The CSF in the lateral ventricles was employed as the zero-susceptibility reference, with the CSF mask generated by thresholding the R2* map computed from mGRE magnitude data and refined using voxel connectivity criteria.

Based on subject-specific binary WMH masks, we examined quantitative susceptibility changes in surrounding white matter regions. Distance maps were generated from the WMH masks using the distancemap tool in FSL (v6.0.5.1), defining three concentric distance-based masks: regions within 0–2 mm, 2–4 mm, and 4–6 mm from the WMH boundary. These distance masks were subsequently intersected with individual white matter masks derived from each subject's segmented T1-weighted image, generated from smoothed probability maps and thresholded at 50 % for white matter inclusion. For each distance mask, mean QSM values were calculated. To evaluate QSM values in the rest of white matter, a mask excluding the 0–6 mm region was created to isolate the remaining white matter (Figure S2).

Furthermore, to explore the affected tracts at the systemic level, we registered the WMH mask to the standard MNI2009c space using the nonlinear deformation field obtained from fMRIPrep. We then employed the WM parcellation defined by the probabilistic tract atlas, Xtracts, to investigate the distribution of affected tracts within areas with WMHs. Forty-two probabilistic tract atlases (see details on the list in Table S1), derived from 1021 subjects in the Human Connectome Project, were used [26].

After diffusion image acquisition, FMRIB software (FSL, v5.09, http://www.fmrib.ox.ac.uk/fsl) was used for DTI processing. Using FSL's eddy-correction tool, eddy current distortions and motion artifacts were corrected by applying affine alignment of each DWI volume to the b0 image. Brain masks were generated from each b0 image using FMRIB's brain extraction tool (BET v2.1) to exclude nonbrain voxels from further consideration. We then used the eddy-corrected 4D data and corresponding brain mask to fit the diffusion tensor model at each voxel by using the FMRIB diffusion toolbox (FDT v3.0). Eigenvalues of the diffusion tensor matrix (λ1, λ2, λ3) were obtained, and maps of axial diffusivity (AD = λ1), mean diffusivity (MD = (λ1 + λ2 + λ3)/3), and fractional anisotropy (FA) were generated. The radial diffusivity (RD, λ23 = (λ2 + λ3)/2) was calculated by averaging the λ2 and λ3 maps. DTI analysis was performed to provide complementary assessment of white matter microstructural integrity alongside QSM-based susceptibility mapping.

### 2.6 Statistical analysis

All the statistical analyses were performed using SPSS software (SPSS; version 26.0 for Windows). P value of 0.05 or less was considered statistically significant, and P-values were FDR-adjusted for multiple comparisons. The distribution of each dataset was checked for normality. Quantitative data are represented as the mean ± standard deviation or median with interquartile range, depending on their distribution, and categorical data are expressed as n ( %). The paired rank sum test was utilized to compare the differences in susceptibility values between WMH areas and NAWM. Non-parametric tests compared the susceptibility values of WMH across the CSVD (0), CSVD (1), and CSVD (≥2) groups. Multivariate logistic regression, adjusting for the ratio of WMH volume to total brain volume, was conducted to analyze the relationship between WMH susceptibility values and variables identified in univariate analyses. After adjusting for confounders such as CSVD severity and the ratio of WMH volume to total brain volume, partial correlation analysis was used to examine the correlations between WMH susceptibility values and cognitive function tests, including the MoCA, AVLT, SDMT, SCWT, and TSUM, and P-values were FDR-adjusted for multiple comparisons. The Mann‒Whitney U test was used to assess differences in WMH volume in forty-two fiber tracts between the CSVD (1) and CSVD (0) groups and between the CSVD (≥2) and CSVD (0) groups. One-way ANOVA was used to compare differences in parameters such as AD, FA, RD, and AD among the CSVD (1), CSVD (≥2), and CSVD (0) groups. For perilesional region analysis, susceptibility values were compared across different distance zones (WMH, 0–2 mm, 2–4 mm, 4–6 mm, and remaining white matter) using the Friedman test for overall comparison, followed by Wilcoxon signed-rank tests for pairwise comparisons between regions. Correlations between regional susceptibility values and cognitive function were examined using partial correlation analysis, controlling for CSVD severity and WMH volume ratio. For longitudinal analysis, linear mixed-effects models were used to examine the relationship between QSM values and cognitive function over time, with participants as random intercepts and adjustment for relevant covariates including age, gender, vascular risk factors, and WMH volume.

## 3 Results

### 3.1 Participant characteristics

Table S2 presents the basic characteristics of the 299 participants included in the study, including data from the QSM, measurements of brain volume, biochemical indicators, demographic data, and neuropsychological test results. The section on brain volume includes statistics for the volume of WMH, total brain volume, and volumes of gray and white matter. In the biochemical markers section, a statistical description of the concentrations of Aβ1–42, total tau (t-Tau), and phosphorylated tau at threonine 181 (p-Tau181) is provided. The demographic data were analyzed for categorical variables: out of 299 participants, 153 were male, representing 51.20 % of the sample. Age was divided into four groups: ≤50 years (64 participants, 21.40 %), ≤60 years (104 participants, 34.80 %), ≤70 years (98 participants, 32.80 %), and ≥71 years (33 participants, 11.00 %). The results of neuropsychological tests, including the MoCA, AVLT, SDMT, SCWT, and TMT, were also statistically described (Table S3). Demographic and other categorical variables are expressed as percentages. The data are presented as the mean ± standard deviation or median with interquartile range, based on the distribution of the data. Of the 364 individuals initially invited, 50 were excluded due to duplicate data from follow-ups, 1 for multiple sclerosis, 6 for image quality issues, 7 for lack of relevant clinical data, and 1 for both multiple sclerosis and poor image quality, resulting in a final sample size of 299 participants (Figure S3).

When participants were stratified by WMH burden based on Fazekas scoring (Table S4), significant differences were observed across groups. Participants with greater WMH burden were significantly older (Fazekas 0: 53.0 [43.8, 60.0] years vs Fazekas 3: 66.0 [61.0, 71.0] years, *P* < 0.001) and demonstrated progressively worse cognitive performance across all domains: MoCA (26.0 [24.0, 28.0] vs 24.0 [20.0, 25.0], *P* < 0.001), AVLT (58.0 [51.0, 73.0] vs 52.0 [42.0, 61.0], *P* < 0.001), SDMT (45.0 [35.5, 56.0] vs 26.0 [17.0, 33.0], *P* < 0.001), and SCWT (110.0 [95.0, 137.0] vs 138.0 [124.0, 199.0], *P* < 0.001). Notably, plasma AD biomarkers (Aβ1–42, total tau, p-Tau181) did not differ significantly across WMH burden groups (all *P* > 0.05).

However, when examining the relationship between plasma biomarkers and QSM-derived susceptibility values within WMH regions (Table S5), total tau showed significant positive associations with WMH core susceptibility (β = 0.52, FDR-adjusted *P* = 0.029) and the immediate 0–2 mm perilesional zone (β = 0.443, FDR-adjusted *P* = 0.041), after adjusting for age, sex, and education. No significant associations were observed for Aβ1–42 or p-Tau181 with QSM values in any region (all FDR-adjusted *P* > 0.05).

### 3.2 Paired rank sum test for susceptibility values of WMH and NAWM

**Fig. 1:**
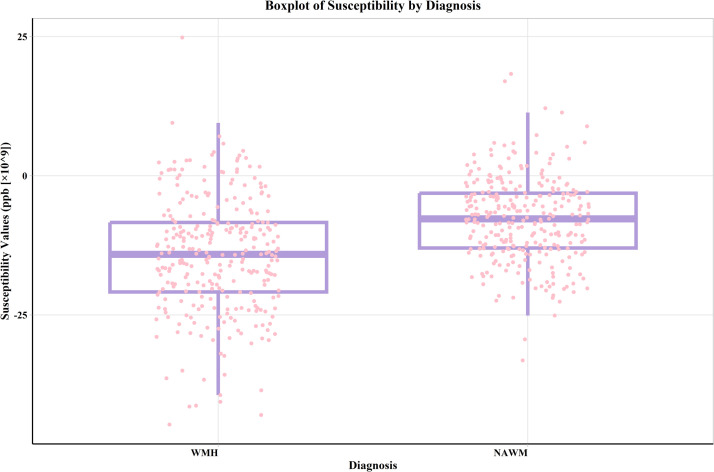
Boxplot of susceptibility values for WMH and NAWM. The susceptibility values of WMH are significantly lower than those of NAWM. Each boxplot's horizontal line indicates the median, while the box edges represent the interquartile range (IQR). *** = *P* ≤ 0.001. Abbreviations: WMH = white matter hyperintensities, NAWM = normal appearing white matter.

Fig. 1 details the differential analysis of the susceptibility values for WMH and NAWM. The distributions of the susceptibility values are presented as medians and interquartile ranges. The findings revealed a significant difference in susceptibility values between WMH and NAWM (*Z* = −11.57, *P* < 0.001). This notable difference indicates that the susceptibility values in WMH areas have experienced significant changes compared to those in NAWM areas, likely reflecting the pathophysiological changes in these areas.

### 3.3 Differential analysis of WMH susceptibility values across the three groups

**Fig. 2:**
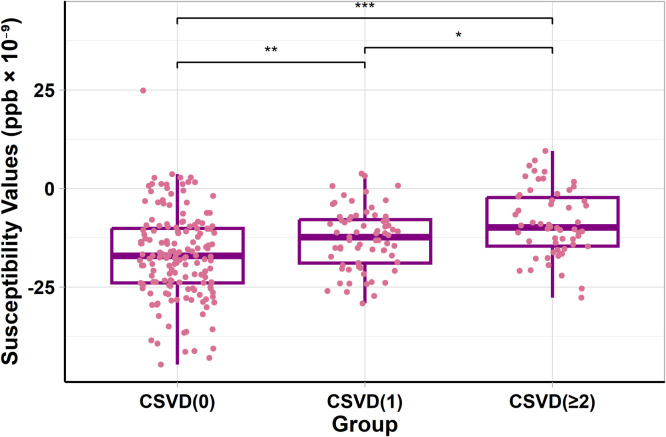
The boxplot shows the distribution and differences in susceptibility values of WMH among CSVD (0), CSVD (1), CSVD (≥2) groups. It reveals that in more severe CSVD cases, the susceptibility values of WMH are higher, with significant differences in pairwise comparisons among the three groups. The horizontal line in each boxplot represents the median, and the box edges correspond to the interquartile range (IQR). * = *P* ≤ 0.05, ** = *P* ≤ 0.01, *** = *P* ≤ 0.001. Abbreviations: CSVD (0) = Score for burden of CSVD = 0, CSVD (1) = Score for burden of CSVD = 1, CSVD (≥2) = Score for burden of CSVD = 2,3,4. CSVD = cerebral small vessel disease. ppb = parts per billion.

There was a significant difference in the susceptibility values of WMH across the three groups (*P* < 0.001) (Fig. 2). After adjustment, the pairwise comparisons remained significant (CSVD (0) vs. CSVD (1): *P* = 0.004; CSVD (0) vs. CSVD (≥2): *P* = 0.001; CSVD (1) vs. CSVD (≥2): *P* = 0.02) (Table S6).

### 3.4 Spatial analysis of susceptibility values in WMH and perilesional regions

Our analysis revealed a distinct spatial pattern in susceptibility values from WMH cores to perilesional white matter regions. In the overall cohort, we observed a U-shaped spatial gradient: susceptibility values were −14.74 ppb in WMH cores, decreased to −16.78 ppb at 0–2 mm (the lowest values among all regions), then progressively increased to −16.28 ppb at 2–4 mm and −15.25 ppb at 4–6 mm, before sharply rising to −7.59 ppb in the remaining white matter beyond 6 mm (Table S7). Friedman test confirmed significant overall differences across these regions (*P* < 0.001). Post-hoc pairwise comparisons demonstrated significant differences between all region pairs (all *P* ≤ 0.002), with susceptibility values progressively increasing (becoming less negative) from the 0–2 mm to 4–6 mm perilesional zones (Table S8).

When stratified by CSVD severity (Table 1), WMH core susceptibility values showed significant differences across groups (*P* < 0.001), becoming progressively less negative with increasing disease severity. The immediate perilesional zone (0–2 mm) showed a similar but attenuated pattern (*P* = 0.009). In contrast, all regions beyond 2 mm from WMH borders showed no significant differences across CSVD groups (2–4mm: *P* = 0.082; 4–6mm: *P* = 0.342; extended analysis to 6–8mm: *P* = 0.512; 8–10mm: *P* = 0.623; >10mm: *P* = 0.687) (Table S9, Figure S4).

**Table 1:** Comparative analysis of susceptibility values in different regions.

Test Variable	**CSVD (0) (*n*=137)**	**CSVD (1) (*n*=62)**	**CSVD (≥2) (*n*=47)**	**P**	**Pair-Wise Test**
WMH	−18.08 (−25.33, −9.63)	−13.04 (−19.42, −8.19)	−11.17 (−16.49, −5.92)	<0.001	.003ᵃ <0.001ᵇ 0.028ᶜ
0–2 mm	−19.18 (−26.04, −10.89)	−15.16 (−19.59, −10.74)	−15.27 (−19.75, −8.01)	.009	.008ᵃ 0.003ᵇ 0.352ᶜ
2–4 mm	−17.31 (−24.43, −10.85)	−15.72 (−21.21, −9.68)	−14.50 (−19.74, −7.95)	.082	.098ᵃ 0.112ᵇ 0.589ᶜ
4–6 mm	−15.70 (−21.31, −9.29)	−14.95 (−20.12, −8.91)	−13.64 (−19.79, −7.65)	.342	.204ᵃ 0.156ᵇ 0.682ᶜ
The rest of WM	−7.59 (−12.82, −3.10)	−7.01 (−13.54, −2.47)	−7.95 (−14.85, −4.15)	.328	.186ᵃ 0.165ᵇ 0.602ᶜ

Note. —Pair-wise test: *a* = CSVD (1) vs. CSVD (0), *b* = CSVD (≥2) vs. CSVD (0), *c* = CSVD (1) vs. CSVD (≥2). CSVD (0) = Score for burden of CSVD = 0, CSVD (1) = Score for burden of CSVD = 1, CSVD (≥2) = Score for burden of CSVD = 2,3,4. CSVD = cerebral small vessel disease, WMH = white matter hyperintensities. 0–2 mm, 2–4 mm, and 4–6 mm represent the regions at different distances from the WMH edge (0–2 mm , 2–4 mm , and 4–6 mm from the WMH border, respectively). The rest of WM represents normal-appearing white matter beyond 6 mm from WMH. Data are expressed as median (interquartile range). P values are from Kruskal-Wallis test. Pairwise comparisons were performed using the Mann-Whitney U test, and P values were false discovery rate (FDR) corrected.

### 3.5 Univariate analysis of WMH susceptibility values

Univariate analyses were conducted as a preliminary screening step to identify candidate variables for subsequent multivariable modeling (Table S10). Factors such as gender, BMI, hyperlipidemia, smoking status, alcohol consumption status, and APOE ε4 were not significantly associated with WMH susceptibility (all *P* > 0.05).

Age was significantly associated with WMH susceptibility values (*Z* = 18.661, *P* < 0.001), with older participants showing progressively higher (less negative) values: ≤50 years [−22.20 (−27.80, −11.74) ppb], ≤60 years [−14.21 (−18.91, −7.03) ppb], ≤70 years [−12.20 (−19.61, −8.65) ppb], and ≥71 years [−11.27 (−17.94, −4.87) ppb]. Hypertension was significantly associated with WMH susceptibility (*Z* = 9.110, *P* = 0.003), with hypertensive participants showing higher values [−12.63 (−17.04, −6.86) ppb] compared to non-hypertensive participants [−15.99 (−23.43, −9.35) ppb]. Similarly, diabetes was associated with higher susceptibility values (*Z* = 13.174, *P* < 0.001): diabetic participants [−11.19 (−17.59, −6.11) ppb] versus non-diabetic participants [−16.65 (−23.57, −9.67) ppb]. Education level (*Z* = 6.138, *P* = 0.046) and CSVD severity (*Z* = 28.780, *P* < 0.001) were also significantly associated with WMH susceptibility values.

### 3.6 Multivariate logistic regression analysis of WMH susceptibility values

Table S11 presents the results of a multivariate logistic regression analysis on the susceptibility values of WMH. Variables that showed significant associations in the univariate analysis (age, hypertension, diabetes, education level, and CSVD severity) were included in the model, with the ratio of WMH volume to total brain volume controlled as a covariate. After adjustment for potential confounders, only CSVD severity and diabetes remained independently associated with WMH susceptibility values. CSVD severity showed a strong independent association, with CSVD(0) participants having significantly lower odds of higher susceptibility values compared to CSVD(≥2) participants (OR= 0.27, *P* = 0.001). Diabetes was also independently associated with WMH susceptibility (OR = 0.59, *P* = 0.047). In contrast, age, hypertension, and education level—all significant in univariate analyses—were no longer significantly associated with WMH susceptibility after adjustment (age: all *P* > 0.33; hypertension: *P* = 0.34; education: all *P* > 0.75).

### 3.7 Partial correlation analysis between WMH susceptibility values and cognitive functions

Table S12 shows the results of partial correlation analyses between WMH susceptibility values and various cognitive function tests, and P-values were FDR-adjusted for multiple comparisons. We analyzed the relationships with five neuropsychological tests, the MoCA, AVLT, SDMT, SCWT, and TSUM, controlling for the severity of CSVD and the ratio of WMH volume to total brain volume. After adjustment, a significant correlation was found between MoCA scores and WMH susceptibility values (MoCA: *r* = −0.155, *P* = 0.045), suggesting that an increase in WMH susceptibility values is associated with a decline in cognitive function (Figure S5). However, the correlations between WMH susceptibility values and other tests, such as the AVLT, SDMT, SCWT, and TSUM, were not significant (AVLT: *r* = −0.092, *P* = 0.22; SDMT: *r* = −0.084, *P* = 0.21; SCWT: *r* = 0.110, *P* = 0.17; TSUM: *r* = 0.069, *P* = 0.25) (Figure S5).

### 3.8 Spatial analysis of correlations between regional susceptibility values and cognitive function

We further analyzed the spatial pattern of correlations between susceptibility values and cognitive function across different white matter regions, including concentric perilesional zones (0–2 mm, 2–4 mm, and 4–6 mm from WMH boundary) and the remaining NAWM. A gradient pattern emerged in these correlations: the strongest correlation was observed in the immediate perilesional zone (0–2 mm) with MoCA scores (*r* = −0.160, uncorrected *p* = 0.042), followed by progressively weaker correlations in more distant regions (2–4mm: *r* = −0.126, *p* = 0.144; 4–6mm: *r* = −0.111, *p* = 0.219) and the remaining NAWM (*r* = −0.110, *p* = 0.228) (Table S13). Similar spatial gradients were observed for other cognitive measures. However, after FDR correction for multiple comparisons, none of these correlations reached statistical significance (all FDR-corrected *p* > 0.122).

### 3.9 Longitudinal analysis of WMH susceptibility values and cognitive function

Linear mixed-effects models examined the longitudinal relationship between WMH susceptibility values and cognitive performance in 71 CSVD participants over a mean follow-up period of 20.6 months. WMH susceptibility values showed a significant negative association with information processing speed as measured by SDMT (β = −0.247, *P* = 0.042), indicating that higher susceptibility values were associated with poorer performance on cognitive processing tasks. Spatial gradient analysis revealed differential longitudinal effects across perilesional regions. The association between susceptibility values and SDMT performance was strongest in WMH core regions (β = −0.247, *P* = 0.042), while becoming non-significant in perilesional zones (0–2mm: β = −0.234, *P* = 0.080; 2–4mm: β = −0.146, *P* = 0.409; 4–6mm: β = −0.002, *P* = 0.991). No significant longitudinal associations were found between WMH susceptibility values and other cognitive measures (MoCA, AVLT, SCWT, TSUM) (Table 2).

**Table 2:** Longitudinal associations between regional QSM values and cognitive function.

**Cognitive Test**	**Region**	**β**	**SE**	**t-value**	**P-value**	**95 % CI**
**AVLT**						
	WMH Core	−0.291	0.155	−1.878	0.063	[−0.595, 0.014]
	0–2mm	−0.148	0.158	−0.935	0.352	[−0.458, 0.163]
	2–4mm	−0.291	0.222	−1.312	0.192	[−0.729, 0.147]
	4–6mm	−0.287	0.267	−1.076	0.284	[−0.814, 0.240]
**SDMT**						
	WMH Core	**−0.247**	**0.120**	**−2.059**	0.042*	**[−0.483, −0.010]**
	0–2mm	−0.234	0.133	−1.768	0.080	[−0.495, 0.027]
	2–4mm	−0.146	0.176	−0.829	0.409	[−0.492, 0.201]
	4–6mm	−0.002	0.211	−0.012	0.991	[−0.419, 0.414]
**MoCA**						
	WMH Core	−0.036	0.034	−1.031	0.305	[−0.104, 0.033]
	0–2mm	0.033	0.034	0.967	0.336	[−0.034, 0.100]
	2–4mm	0.045	0.049	0.919	0.360	[−0.052, 0.142]
	4–6mm	0.046	0.059	0.772	0.441	[−0.072, 0.164]
**SCWT**						
	WMH Core	0.398	0.472	0.843	0.401	[−0.533, 1.329]
	0–2mm	−0.021	0.518	−0.041	0.967	[−1.043, 1.001]
	2–4mm	−0.383	0.687	−0.557	0.578	[−1.740, 0.974]
	4–6mm	−0.365	0.817	−0.446	0.656	[−1.979, 1.250]
**TSUM**						
	WMH Core	0.948	1.252	0.757	0.451	[−1.525, 3.421]
	0–2mm	−0.392	1.363	−0.288	0.774	[−3.081, 2.297]
	2–4mm	−2.511	1.801	−1.395	0.166	[−6.070, 1.048]
	4–6mm	−3.484	2.124	−1.640	0.104	[−7.685, 0.717]

Linear mixed-effects models examined the relationship between regional quantitative susceptibility mapping (QSM) values and cognitive performance in 71 cerebral small vessel disease participants over a mean follow-up of 20.6 months. Models included participants as random intercepts with adjustment for age, gender, smoking status, drinking status, hypertension, white matter hyperintensity volume, and remaining white matter QSM values.

### 3.10 Univariate anova of DTI parameters across different groups

**Fig. 3:**
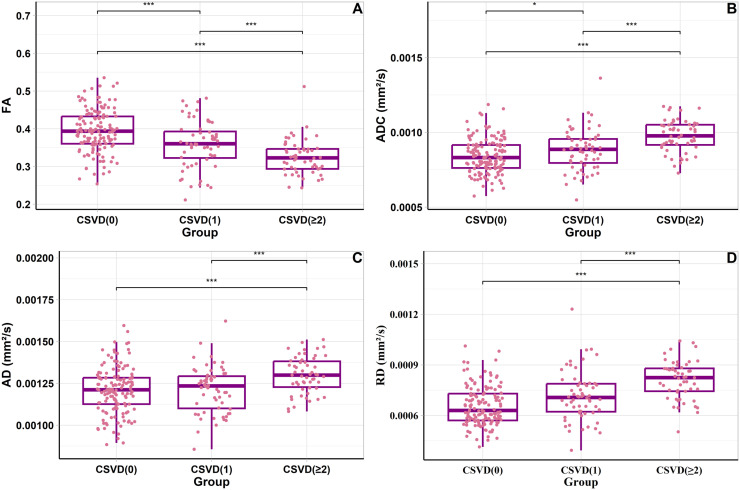
Box plots depicting the variation in four DTI imaging parameters **(A, B, C, D)** across the spectrum of CSVD. FA values are significantly lower in the CSVD (≥2) group compared to CSVD (0) **(A)**, while RD, AD, ADC values are significantly higher **(B, C, D)**. Comparing CSVD (≥2) and CSVD (1) groups, FA values remain significantly lower in the CSVD (≥2) group **(A)**, and RD, AD, ADC values are higher **(B, C, D)**. Only AD shows no significant difference when comparing CSVD (1) with CSVD (0) **(C)**. Horizontal lines in each box plot represent the median, and the box lines correspond to the interquartile range (IQR). * = *P* ≤ 0.05, ** = *P* ≤ 0.01, *** = *P* ≤ 0.001. Abbreviations: CSVD (0) = score for burden of CSVD = 0, CSVD (1) = Score for burden of CSVD = 1, CSVD (≥2) = score for burden of CSVD = 2,3,4, CSVD = cerebral small vessel disease, DTI = diffusion tensor imaging, FA = fractional anisotropy, RD = radial diffusivity, ADC = apparent diffusion coefficient, AD = axial diffusivity.

To characterize white matter microstructural integrity alongside susceptibility changes, we performed DTI analysis across CSVD severity groups (Fig. 3, Figure S6 and Table S14). Compared to CSVD(0), the CSVD(≥2) group showed significantly reduced FA (0.33 ± 0.05 vs 0.40 ± 0.06, *P* < 0.001) and elevated RD, AD, and ADC values (all *P* < 0.001). The CSVD(1) group showed intermediate values for most parameters. AD did not differ significantly between CSVD(0) and CSVD(1) (*P* = 0.87), but differed significantly between CSVD(1) and CSVD(≥ 2) (*P* < 0.001).

### 3.11 Statistical analysis of WMH volume changes in white matter fiber tracts during CSVD progression

We measured WMH volumes across 42 white matter fiber tracts by performing differential tests between the CSVD (0) and CSVD (1) groups and between the CSVD (0) and CSVD (≥2) groups, and the corresponding *P*-values were calculated. To mitigate the risk of false positives from multiple comparisons, *P*-values were FDR-adjusted. In comparisons between CSVD (0) and CSVD (1), several fiber tracts demonstrated statistically significant differences (Table S15). Notable differences were observed in tracts such as the Anterior Commissure (*Z* = −2.111, *P* = 0.049), Arcuate Fasciculus L (*Z* = −5.554, *P* < 0.001), and Cingulum subsection Dorsal L (*Z* = −3.377, *P* = 0.002) tracts. In the analysis between CSVD (0) and CSVD (≥2), almost all tracts showed significant differences, indicating the potential impact of disease severity (Figure S7). Particularly pronounced differences were noted in tracts such as the Arcuate Fasciculus R (*Z* = −8.916, *P* < 0.001) and Corticospinal Tract R (*Z* = −10.015, *P* < 0.001). These findings suggest significant variations in WMH volumes in white matter fiber tracts among individuals with varying severities of CSVD.

## 4 Discussion

Our multimodal neuroimaging approach integrates QSM and DTI to characterize white matter pathology in CSVD. QSM quantifies tissue magnetic susceptibility, reported in parts per billion (ppb), with higher (less negative) values indicating paramagnetic iron accumulation and lower (more negative) values suggesting diamagnetic myelin content or iron depletion. DTI assesses microstructural integrity through water diffusion patterns: FA measures fiber tract organization (range 0–1), with higher values reflecting intact white matter structure and lower values indicating disruption; RD and AD measure perpendicular and parallel diffusion respectively, with elevated values suggesting tissue degradation. The combination of progressively increasing WMH susceptibility values and declining FA across CSVD severity groups demonstrates that iron dysregulation occurs alongside microstructural breakdown, contributing to white matter damage and cognitive decline.

QSM is broadly applied in brain research, especially for gray matter iron dysregulation, but its role in delineating changes in WMH susceptibility values in CSVD and its relationship with cognitive functions remain unclear. This study revealed a statistically significant reduction in susceptibility values in WMHs compared to those in NAWMs (*P* < 0.001). There were significant differences in WMH susceptibility values among the CSVD (0), CSVD (1), and CSVD (≥2) groups (*P* < 0.001). In more severe CSVD cases, the susceptibility values of WMH are relatively greater. A negative correlation between WMH susceptibility values and MoCA scores (*r* = −0.12; *P* = 0.045) was found. DTI parameters such as ADC, FA, RD, and AD showed significant differences at various stages of CSVD (ADC: *P* < 0.001; FA: *P* < 0.001; RD: *P* < 0.001; AD: *P* < 0.001).

Simultaneous iron accumulation and demyelination are indistinguishable when employing QSM alone, given that both processes lead to increased susceptibility signals [13]. Therefore, our results likely reflect the combined effects of demyelination and iron accumulation within WMH regions. The significant changes observed in FA and RD values across the three CSVD groups provide further insights into the progression of demyelination [27]. Specifically, FA reductions and RD elevations are indicative of impaired myelin integrity and microstructural disorganization, aligning with the notion that CSVD is associated with progressive myelin damage [27]. As CSVD severity increases, endothelial dysfunction and blood-brain barrier (BBB) damage exacerbate myelin degradation and impede its repair [28]. Degraded myelin debris, derived from oligodendrocytes, is phagocytosed by microglia, which selectively accumulate ferritin light chain [3], a protein that binds iron. This process likely explains the increased WMH susceptibility values observed with disease progression, as QSM is highly sensitive to iron content in ferritin.

Interestingly, WMH susceptibility values were found to be significantly lower than those of NAWM. We hypothesize that this phenomenon may be attributable to the disruption and maturation anomalies of oligodendrocytes, which are notably rich in ferritin. Oligodendrocytes not only produce myelin but also regulate iron homeostasis, and their dysfunction can result in impaired iron homeostasis and reduced diamagnetic myelin content. Dysfunctional endothelial cells further compound this problem by inhibiting oligodendrocyte precursor cell maturation, thereby impeding myelin formation and repair [29]. Consequently, endothelial cell dysfunction, a hallmark of early CSVD, may underlie the observed differences in susceptibility values between WMH and NAWM. Our finding aligns with recent clinical trials where iron chelation therapy worsened cognitive outcomes [30], supporting the concept that iron depletion rather than accumulation may characterize early white matter pathology.

Our findings suggest that the pathophysiological changes in WMH in CSVD can be further elucidated by integrating QSM and DTI results. QSM primarily highlights iron accumulation and myelin loss, while DTI provides complementary insights into microstructural integrity [8,14]. For instance, in the early stages of CSVD (CSVD 0), FA remains relatively preserved, and RD shows minimal elevation, suggesting that myelin integrity is largely intact. However, as the disease progresses to moderate stages (CSVD 1), significant FA reductions and RD elevations indicate early demyelination and microstructural disruption. In severe CSVD (CSVD ≥2), these changes become more pronounced, reflecting extensive demyelination and loss of white matter integrity, as corroborated by markedly increased WMH susceptibility values.

Our DTI analysis (Section 3.10) complements the QSM findings by demonstrating concurrent microstructural changes alongside iron dysregulation. The observed reduction in FA (0.33 vs 0.40 in CSVD≥2 vs CSVD0) and elevation in RD, AD, and ADC values reflect progressive demyelination and loss of white matter structural integrity. Notably, the delayed AD changes between CSVD(0) and CSVD(1) suggest that axonal damage becomes prominent primarily in more advanced disease stages. These progressive DTI changes demonstrate that iron-related susceptibility alterations detected by QSM occur within the context of broader microstructural white matter disruption.

The interactions within the neurogliovascular unit (NGVU), comprising endothelial cells, pericytes, astrocytes, oligodendrocytes, and microglia, are critical to understanding these pathophysiological changes. Dysfunctional endothelial cells not only contribute to myelin damage through microvascular dysfunction but also disrupt iron homeostasis by impeding ferritin metabolism [29]. Furthermore, microglial activation in response to myelin debris exacerbates iron dysregulation, forming a feedback loop that accelerates demyelination and susceptibility changes [3]. These findings underscore the importance of a multimodal approach combining QSM and DTI metrics to unravel the complex interplay between demyelination, iron dysregulation, and endothelial dysfunction in CSVD.

The spatial gradient of susceptibility changes in perilesional regions provides insights into WMH pathogenesis. The stability of susceptibility values in outer perilesional zones (2–6 mm) across CSVD severity groups, consistent with myelin water imaging showing no demyelination in these areas [31], suggests these changes primarily reflect iron redistribution rather than structural damage. This spatial pattern may indicate a transitional zone where early iron dysregulation precedes overt tissue injury..

Our study also found a significant correlation between WMH susceptibility values and MoCA scores, suggesting an association between increased WMH susceptibility values and cognitive decline. Iron dysregulationwithin WMH may reflect demyelination and other damages in white matter, potentially affecting neuronal signal transmission and network efficiency, especially in brain networks involved in cognitive processing. Additionally, degenerated microglia can be found in cases of CSVD. The degenerated microglia that have phagocytosed myelin debris might undergo iron-mediated lipid peroxidation damage, a core feature of ferroptosis [3]. Ferroptotic microglia are considered to produce neurotoxicity, leading to neuronal damage [6], which might partly explain the cognitive and motor function impairments in the later stages of CSVD. The change in QSM value is not only due to myelin/axonal loss, iron deposition but also to oligodendrocyte damage and iron loss. QSM value provides a measure that is comprehensively reflecting these brain tissue damages. Therefore, WMH susceptibility values could reflect cognitive decline in CSVD. This phenomenon suggested imbalances in iron homeostasis contributing to neurodegeneration might not be confined to neurons alone but could also, or predominantly, arise in glial cells. Acknowledging the pivotal role of glial cells in iron regulation is crucial for a thorough understanding of iron dynamics within the brain.

Our study provides complementary insights from both cross-sectional and longitudinal analyses. The cross-sectional correlation between WMH susceptibility values and MoCA scores (*r* = −0.12, *P* = 0.045) demonstrates that iron dysregulation within WMH is associated with global cognitive function, while the longitudinal association with SDMT performance (β = −0.247, *P* = 0.042) reveals that these iron-related changes specifically predict decline in information processing speed over time.

This dual pattern suggests different temporal relationships between iron dysregulation and cognitive function: WMH susceptibility values appear to reflect current global cognitive status while also serving as predictors of future decline in specific cognitive domains. The longitudinal association with information processing speed is particularly noteworthy, as SDMT measures a cognitive domain highly sensitive to white matter integrity, supporting the hypothesis that iron dysregulation within WMH directly impact neural signal transmission efficiency over time.

Our findings suggest a mechanistic framework where chronic hypoperfusion triggers oligodendrocyte dysfunction and progressive iron dysregulation, creating a self-amplifying cycle of oxidative injury and demyelination. This framework identifies potential therapeutic targets including cerebral perfusion, oxidative stress mitigation, and iron homeostasis modulation, with QSM and DTI serving as non-invasive biomarkers for monitoring interventions.

Our analysis of plasma AD biomarkers revealed an interesting pattern: while Aβ1–42, total tau, and p-Tau181 concentrations did not differ across WMH burden groups, total tau levels showed significant positive correlations with QSM-derived susceptibility in WMH cores and immediate perilesional zones. This suggests that iron dysregulation may represent a mechanistic link between vascular pathology and neurodegenerative processes, independent of overall WMH volume. The specific association with tau but not amyloid warrants further investigation in future studies examining the interplay between CSVD and Alzheimer's disease pathology.

Several considerations should be noted when interpreting our findings. Our longitudinal analysis, while providing valuable insights into the temporal relationship between WMH susceptibility values and cognitive decline, was conducted over a mean follow-up period of 20.6 months. Extended follow-up studies would further strengthen our understanding of the long-term predictive utility of QSM measurements in CSVD progression. The longitudinal cognitive associations were most clearly observed for information processing speed (SDMT), suggesting potential domain-specificity in the relationship between iron dysregulation and cognitive function. This finding opens important avenues for future investigation in larger cohorts with comprehensive cognitive assessments. While QSM provides valuable information about tissue susceptibility changes, integration with complementary techniques such as DTI enhances interpretation of the underlying pathophysiological processes. Future histopathological correlation studies would provide additional validation of our QSM-based interpretations and further elucidate the contributions of iron dysregulation, demyelination, and glial changes to the observed patterns. Our single-center design ensured standardized acquisition protocols and consistent image quality. Multi-center validation would support the broader applicability of these findings across different populations and imaging platforms. We did not perform CSF sampling or amyloid PET imaging, precluding definitive AD biomarker classification. Therefore, plasma biomarkers were analyzed as continuous variables rather than applying AD-specific cut-offs that may not be validated for CSVD populations. Future studies with CSF or PET confirmation are needed to establish CSVD-specific thresholds. Finally, while our longitudinal design demonstrates temporal associations between WMH susceptibility values and cognitive changes, intervention studies targeting iron homeostasis pathways would provide complementary evidence for causal relationships and therapeutic potential. These considerations do not diminish the significance of our findings but rather highlight opportunities for future research to build upon this foundation.

## 5 Conclusions

Our findings demonstrate that WMH susceptibility values are significantly reduced compared to normal-appearing white matter, with progressive increases correlating with CSVD severity. Spatial analysis revealed distinct patterns of iron dysregulation, with WMH cores showing pronounced severity-dependent changes while distant perilesional regions remained relatively stable. Cross-sectionally, WMH susceptibility values correlate with global cognitive function, while longitudinally they predict decline in information processing speed. These insights underscore the clinical potential of QSM in CSVD research and patient monitoring. Quantitative susceptibility mapping emerges as a promising tool for studying CSVD pathophysiology and identifying patients at risk for cognitive decline in future clinical applications.

## Funding

This work was supported by grants from the 10.13039/501100001809National Natural Science Foundation of China (82,272,072), the 10.13039/501100007129Natural Science Foundation of Shandong Province (ZR2020MH288, ZR2024MH026), the 10.13039/100016099Technology Development Plan of Jinan (202,328,066), Funding for Study Abroad Program by Shandong Province (201,803,059), and the 10.13039/501100010227Medical and Health Science and Technology Development Project of Shandong Province (202,309,010,557; 202,309,010,560; 202,409,010,479), and the Shandong Province Medical System Employee Science and Technology Innovation Plan (SDYWZGKCIH2023034; SDYWZGKCIH2024021).

## Ethical approval

All study procedures were approved by the Ethical Committee of the Institutional Review Board (IRB) of Shandong Institute of Medical Imaging (2019–002). The study was conducted in accordance with the Declaration of Helsinki. All participants signed an informed consent form before the commencement of the study.

## Availability of data and materials

The datasets generated and/or analyzed during the current study are not publicly available due to privacy or ethical restrictions but are available from the corresponding author on reasonable request.

## Ethics approval and consent to participate

All study procedures were approved by the Ethical Committee of the Institutional Review Board (IRB) of Shandong Institute of Medical Imaging (2019–002). The study was conducted in accordance with the Declaration of Helsinki. All participants signed an informed consent form before the commencement of the study.

## Consent for publication

Not applicable.

## CRediT authorship contribution statement

**Pengcheng Liang:** Writing – original draft, Visualization. **Meng Li:** Writing – review & editing, Visualization. **Qihao Zhang:** Software. **Nan Zhang:** Data curation. **Yena Che:** Data curation. **Yian Gao:** Data curation. **Chaofan Sui:** Data curation. **Xinyue Zhang:** Data curation. **Na Wang:** Data curation. **Yuanyuan Wang:** Data curation. **Yiwen Chen:** Data curation. **Zhenyu Cheng:** Data curation. **Changhu Liang:** Writing – review & editing, Funding acquisition. **Lingfei Guo:** Writing – review & editing, Project administration, Funding acquisition, Data curation. **Jing Li:** Writing – review & editing, Funding acquisition.

## Declaration of competing interest

The authors declare that they have no competing interests.

## Appendix Supplementary materials

Image, application 1

Image, application 2

## References

[1] Prins N.D., Scheltens P. (2015). White matter hyperintensities, cognitive impairment and dementia: an update. Nat Rev Neurol.

[2] Wardlaw J.M., Valdés Hernández M.C., Muñoz-Maniega S. (2015). What are white matter hyperintensities made of? Relevance to vascular cognitive impairment. J Am Heart Assoc.

[3] Adeniyi P.A., Gong X., MacGregor E., Degener-O’Brien K., McClendon E., Garcia M. (2023). Ferroptosis of microglia in aging human white matter injury. Ann Neurol.

[4] Górska A., Markiewicz-Gospodarek A., Markiewicz R., Chilimoniuk Z., Borowski B., Trubalski M. (2023). Distribution of iron, copper, zinc and cadmium in glia, their influence on glial cells and relationship with neurodegenerative diseases. Brain Sci.

[5] Bradl M., Lassmann H. (2010). Oligodendrocytes: biology and pathology. Acta Neuropathol.

[6] Ryan S.K., Zelic M., Han Y., Teeple E., Chen L., Sadeghi M. (2023). Microglia ferroptosis is regulated by SEC24B and contributes to neurodegeneration. Nat Neurosci.

[7] Attems J., Jellinger K.A. (2014). The overlap between vascular disease and Alzheimer’s disease–lessons from pathology. BMC Med.

[8] Wang Y., Spincemaille P., Liu Z., Dimov A., Deh K., Li J. (2017). Clinical quantitative susceptibility mapping (QSM) – Biometal imaging and its emerging roles in patient care. J Magn Reson Imaging.

[9] Haacke E.M., Liu S., Buch S., Zheng W., Wu D., Ye Y. (2015). Quantitative susceptibility mapping: current status and future directions. Magn Reson Imaging.

[10] Fiscone C., Rundo L., Lugaresi A., Manners D.N., Allinson K., Baldin E. (2023). Assessing robustness of quantitative susceptibility-based MRI radiomic features in patients with multiple sclerosis. Sci Rep.

[11] Vergoossen L.W.M., Jansen J.F.A., van Sloten T.T., Stehouwer C.D.A., Schaper N.C., Wesselius A. (2021). Interplay of white matter hyperintensities, cerebral networks, and cognitive function in an adult population: diffusion-tensor imaging in the Maastricht study. Radiology.

[12] Howard C.M., Jain S., Cook A.D., Packard L.E., Mullin H.A., Chen N.-K. (2022). Cortical iron mediates age-related decline in fluid cognition. Hum Brain Mapp.

[13] Wiggermann V., Hametner S., Hernández-Torres E., Kames C., Endmayr V., Kasprian G. (2017). Susceptibility-sensitive MRI of multiple sclerosis lesions and the impact of normal-appearing white matter changes. NMR Biomed.

[14] Yu F.F., Chiang F.L., Stephens N., Huang S.Y., Bilgic B., Tantiwongkosi B. (2019). Characterization of normal-appearing white matter in multiple sclerosis using quantitative susceptibility mapping in conjunction with diffusion tensor imaging. Neuroradiology.

[15] Yano R., Hata J., Abe Y., Seki F., Yoshida K., Komaki Y. (2018). Quantitative temporal changes in DTI values coupled with histological properties in cuprizone-induced demyelination and remyelination. Neurochem Int.

[16] Duering M., Biessels G.J., Brodtmann A., Chen C., Cordonnier C., De Leeuw F.-E. (2023). Neuroimaging standards for research into small vessel disease—advances since 2013. Lancet Neurol.

[17] Staals J., Makin S.D.J., Doubal F.N., Dennis M.S., Wardlaw J.M. (2014). Stroke subtype, vascular risk factors, and total MRI brain small-vessel disease burden. Neurology.

[18] Fazekas F., Chawluk J.B., Alavi A., Hurtig H.I., Zimmerman R.A. (1987). MR signal abnormalities at 1.5 T in Alzheimer’s dementia and normal aging. AJR Am J Roentgenol.

[19] Bergeron D., Flynn K., Verret L., Poulin S., Bouchard R.W., Bocti C. (2017). Multicenter validation of an MMSE-MoCA conversion table. J Am Geriatr Soc.

[20] Lu J., Li D., Li F., Zhou A., Wang F., Zuo X. (2011). Montreal cognitive assessment in detecting cognitive impairment in Chinese elderly individuals: a population-based study. J Geriatr Psychiatry Neurol.

[21] Wei M., Shi J., Li T., Ni J., Zhang X., Li Y. (2018). Diagnostic accuracy of the Chinese version of the trail-making test for screening cognitive impairment. J Am Geriatr Soc.

[22] Esteban O., Markiewicz C.J., Blair R.W., Moodie C.A., Isik A.I., Erramuzpe A. (2019). fMRIPrep: a robust preprocessing pipeline for functional MRI. Nat Methods.

[23] Andermatt S., Pezold S., Cattin P.C., Crimi A., Bakas S., Kuijf H., Menze B., Reyes M. (2018). Brainlesion: glioma, multiple sclerosis, stroke and traumatic brain injuries.

[24] Liu Z., Spincemaille P., Yao Y., Zhang Y., Wang Y. (2018). MEDI+0: morphology enabled dipole inversion with automatic uniform cerebrospinal fluid zero reference for quantitative susceptibility mapping. Magn Reson Med.

[25] Liu T., Khalidov I., De Rochefort L., Spincemaille P., Liu J., Tsiouris A.J. (2011). A novel background field removal method for MRI using projection onto dipole fields (PDF). NMR Biomed.

[26] XTRACT - standardised protocols for automated tractography in the human and macaque brain - PubMed n.d. https://pubmed.ncbi.nlm.nih.gov/32407993/(accessed May 7, 2024). 10.1016/j.neuroimage.2020.116923 PMC7260058 32407993

[27] Kronlage M., Pitarokoili K., Schwarz D., Godel T., Heiland S., Yoon M.-S. (2017). Diffusion tensor imaging in chronic inflammatory demyelinating polyneuropathy: diagnostic accuracy and correlation with electrophysiology. Invest Radiol.

[28] Quick S., Moss J., Rajani R.M., Williams A. (2021). A vessel for change: endothelial dysfunction in cerebral small vessel disease. Trends Neurosci.

[29] Rajani R.M., Quick S., Ruigrok S.R., Graham D., Harris S.E., Verhaaren B.F.J. (2018). Reversal of endothelial dysfunction reduces white matter vulnerability in cerebral small vessel disease in rats. Sci Transl Med.

[30] Ayton S., Barton D., Brew B., Brodtmann A., Clarnette R., Desmond P. (2025). Deferiprone in Alzheimer Disease: a randomized clinical trial. JAMA Neurol.

[31] Ferris J.K., Greeley B., Vavasour I.M., Kraeutner S.N., Rinat S., Ramirez J. (2022). In vivo myelin imaging and tissue microstructure in white matter hyperintensities and perilesional white matter. Brain Commun.

