# Supplementary material for: Iron dysregulation in cerebral small vessel disease: A quantitative susceptibility mapping study revealing spatial patterns and cognitive predictive value
Source: J Prev Alzheimers Dis. 2026 Jan 1;13(2):100451. doi: 10.1016/j.tjpad.2025.100451 (PMC12869043; doi:10.1016/j.tjpad.2025.100451)
Supplement: Supplementary file 1 [file mmc1.pdf]

ISRCTN13222678 https://doi.org/10.1186/ISRCTN13222678

Neuroimaging research of brain aging in the elderly in the community

Submission date	Recruitment status	
20/02/2024	Recruiting	✓ Retrospectively registered
		? Protocol not yet added
Registration date	Overall study status	? SAP not yet added
28/02/2024	Ongoing	– Results not yet expected
Last Edited	Condition category	– Raw data not yet expected
28/02/2024	Nervous System Diseases	✓ Record updated in last year

Plain English Summary
Background and study aims
This project seeks to investigate how the aging process affects the brain and its impact on cognitive abilities and physical movement in adults who are middle-aged and elderly. Additionally, it aims to understand the mechanisms that connect changes in the aging brain to alterations in mental health and cognitive function. By doing so, it aims to enhance our ability to diagnose and predict conditions related to brain aging and to pinpoint potential treatments.

Who can participate?
All subjects aged 40 - 70 years

What does the study involve?
The research includes evaluating how people think and move, testing blood for various substances, and taking images of the brain using a technique called quantitative susceptibility mapping (QSM).

What are the possible benefits and risks of participating?
None

Where is the study run from?
Shandong Provincial Hospital Affiliated to Shandong First Medical University (China)

When is the study starting and how long is it expected to run for?
November 2018 to December 2028

Who is funding the study?
Shandong Provincial Hospital Affiliated to Shandong First Medical University (China)

Who is the main contact?
Lingfei Guo, glfsci@163.com

Contact information

Type(s)
Public, Scientific, Principal Investigator

Contact name
Dr Lingfei Guo

ORCID ID
http://orcid.org/0000-0002-4885-625X

Contact details
Shandong Provincial Hospital Affiliated to Shandong First Medical University
Jinan
China
250021
+86 531-68776789
guolingfei@sdfmu.edu.cn

Additional identifiers

EudraCT/CTIS number
Nil known

IRAS number

ClinicalTrials.gov number
Nil known

Protocol/serial number
Nil known

Study information

Scientific Title
Neuroimaging study on the correlation between brain aging and cognitive-motor function in the middle-aged and elderly population

Study hypothesis
This project aims to explore the correlation between brain aging and cognitive-motor function in the middle-aged and elderly population and to elucidate pathways from age-related brain changes to neuropsychiatric changes, improving diagnosis and prognosis and identifying therapeutic targets.

Ethics approval required
Ethics approval required

Ethics approval(s)
Approved 18/11/2019, Shandong Institute of Medical Imaging (324 Jing-wu Road, Jinan, Shandong, Jinan, 250021, China; +86 68776789; kewaichu@126.com), ref: 2019-002

Study design
Observational cross sectional

Primary study design
Observational

Secondary study design
Cross sectional study

Study setting(s)
Community, Hospital

Study type(s)
Diagnostic, Treatment

Participant information sheet

Condition
Brain aging

Interventions
We intend to utilize the most current Quantitative susceptibility mapping (QSM) data available to examine and evaluate the correlation between iron accumulation and neuropsychiatric disorders. Extensive health data will be utilized to evaluate and compare vascular, inflammatory, metabolic, and genetic risk factors for brain ageing in the elderly and progression through techniques such as regression analyses, and mediation modelling.

Intervention Type
Other

Primary outcome measure
Brain iron is measured using Quantitative susceptibility mapping (QSM) at baseline, 2 years and 5 years.

Secondary outcome measures
Brain iron measured using QSM at 2 years.

Overall study start date
01/11/2018

Overall study end date
01/12/2028

Eligibility

Participant inclusion criteria
1. Age from 40 to 80 years old
2. Right-handedness

Participant type(s)

Healthy volunteer, Patient

Age group
Adult

Lower age limit
40 Years

Upper age limit
80 Years

Sex
Both

Target number of participants
3000

Participant exclusion criteria
1. History of brain trauma, surgery, or tumors
2. Acute complications of type 2 diabetes
3. Severe hypertension
4. History of severe cerebrovascular, neurological, or mental diseases
5. Alcohol or drug abuse
6. MRI contraindications

Recruitment start date
01/12/2018

Recruitment end date
01/11/2028

Locations

Countries of recruitment
China

Study participating centre
Shandong Provincial Hospital Affiliated to Shandong First Medical University
324 Jing-wu Road
Jinan
China
250021

Sponsor information

Organisation
Shandong Provincial Hospital Affiliated to Shandong First Medical University

Sponsor details
324 Jing-wu Road
Jinan
China
250021
+86 531-68776789
guolingfei@sdfmu.edu.cn

Sponsor type
Hospital/treatment centre

Funder(s)

Funder type
Hospital/treatment centre

Funder Name
Shandong Provincial Hospital Affiliated to Shandong First Medical University

Results and Publications

Publication and dissemination plan
Planned publication in a high-impact peer-reviewed journal.

Intention to publish date
01/01/2029

Individual participant data (IPD) sharing plan
The datasets generated during and/or analysedduring the current study will be available upon request from Lingfei Guo, glfsci@163.com

IPD sharing plan summary
Available on request
