# Supplementary material for: Iron dysregulation in cerebral small vessel disease: A quantitative susceptibility mapping study revealing spatial patterns and cognitive predictive value
Source: J Prev Alzheimers Dis. 2026 Jan 1;13(2):100451. doi: 10.1016/j.tjpad.2025.100451 (PMC12869043; doi:10.1016/j.tjpad.2025.100451)
Supplement: Supplementary file 2 [file mmc2.docx]

**Supplementary Materials**

**Figure S1：Workflow for QSM and WMH analysis in this study.**

**
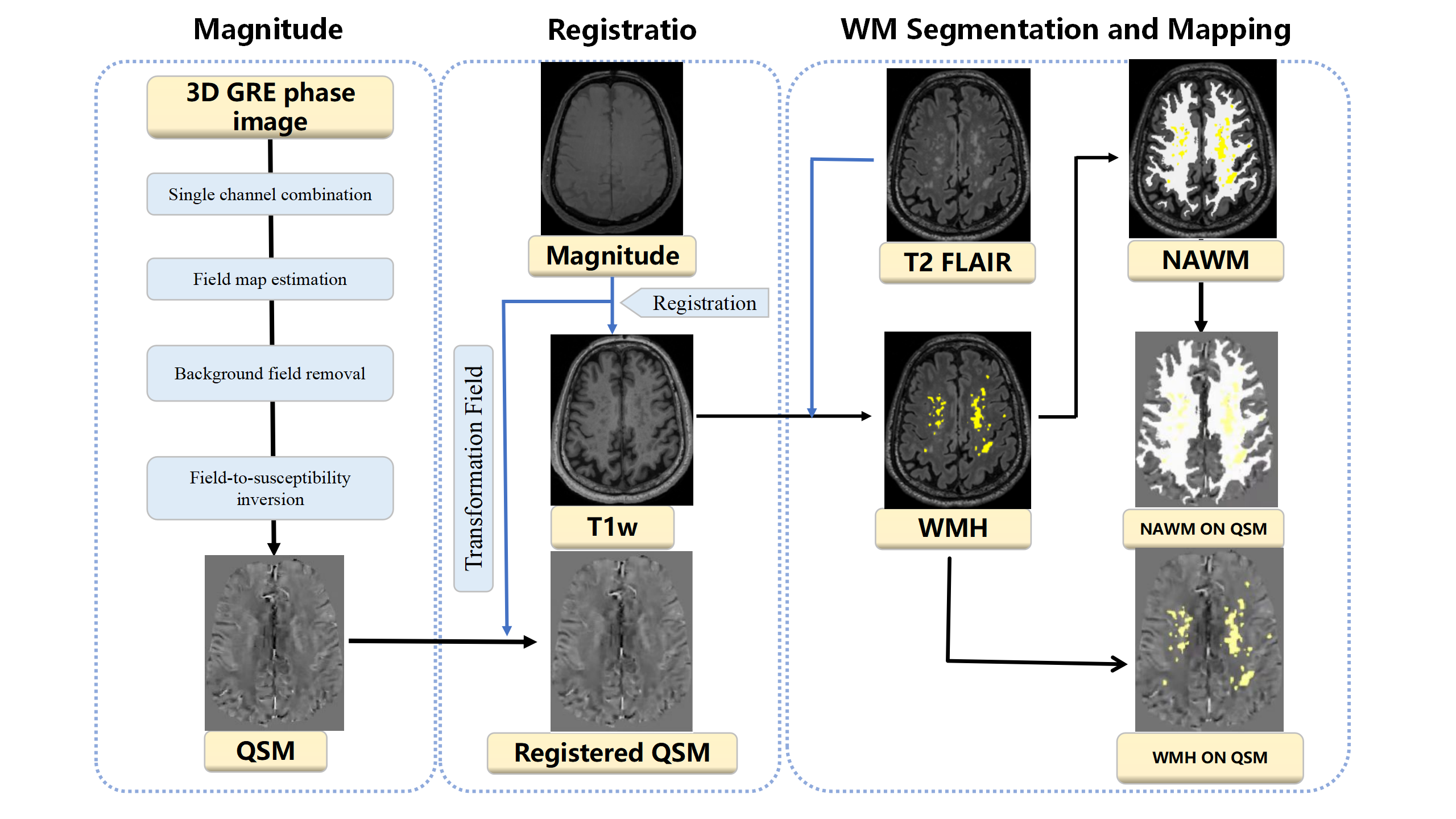
**

This figure illustrates the processing pipeline used for quantitative susceptibility mapping (QSM) and white matter hyperintensity (WMH) analysis. QSM was generated from the 3D GRE phase images through single-channel combination, field map estimation, background field removal, and field-to-susceptibility inversion. Magnitude images were linearly registered to T1-weighted images using FSL's FLIRT algorithm (6 degrees of freedom), and the transformation field was applied to align the QSM data to the native T1 space. For WM segmentation, T2-FLAIR images were used to identify WMH regions, and NAWM masks were created based on an atlas registered to the same space. These masks were then overlaid on the QSM to extract susceptibility values for both WMH and NAWM, ensuring precise spatial alignment and quantification for further analysis.

Abbreviations: QSM = Quantitative Susceptibility Mapping; WMH = White Matter Hyperintensity; NAWM = Normal Appearing White Matter; GRE = Gradient Echo; T1w = T1-weighted image; FLAIR = Fluid-Attenuated Inversion Recovery; FLIRT = FMRIB's Linear Image Registration Tool.

**Figure S2： Spatial analysis framework for examining WMH and perilesional white matter regions.**

**
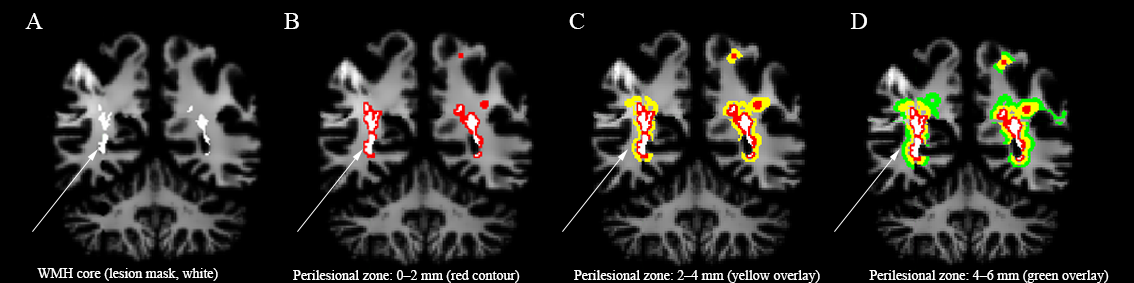
**

(A) Standard brain template showing the WMH core mask (white, indicated by arrow). (B) The immediate perilesional zone (0-2mm from WMH boundary, red contour) surrounding the WMH core (white). (C) Addition of the 2-4mm perilesional zone (yellow overlay). (D) Complete spatial segmentation showing all concentric zones: WMH core (white), 0-2mm zone (red), 2-4mm zone (yellow), and 4-6mm zone (green overlay). Distance maps were generated from WMH masks using FSL's distancemap tool, defining three concentric distance-based regions.

**Figure S3：Flowchart of Participant Selection.**

**
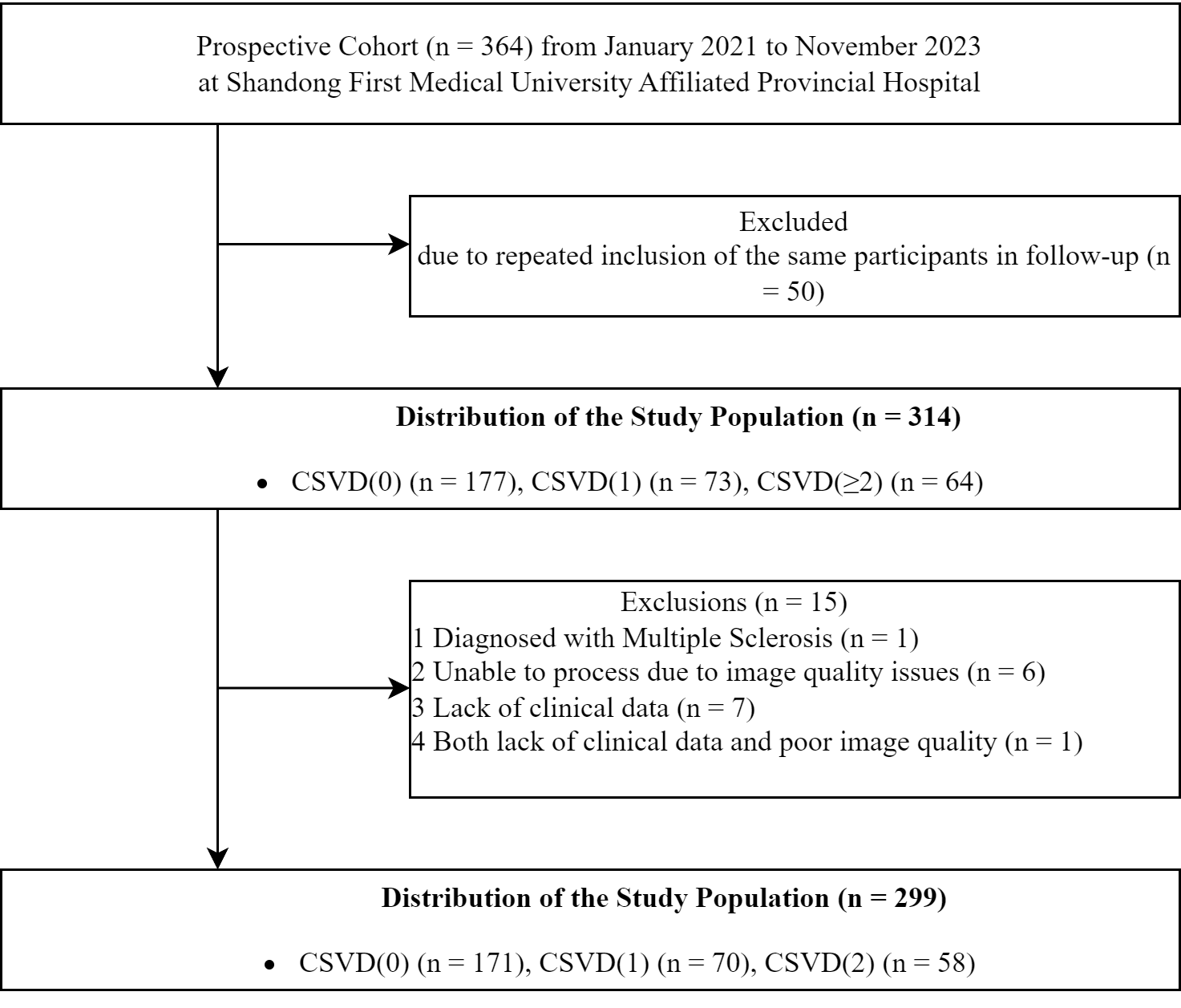
**

CSVD (0) = score for burden of CSVD = 0, CSVD (1) = score for burden of CSVD = 1, CSVD (≥2) = score for burden of CSVD = 2,3,4.

**Figure S4. QSM Values across Regions and CSVD Severity**


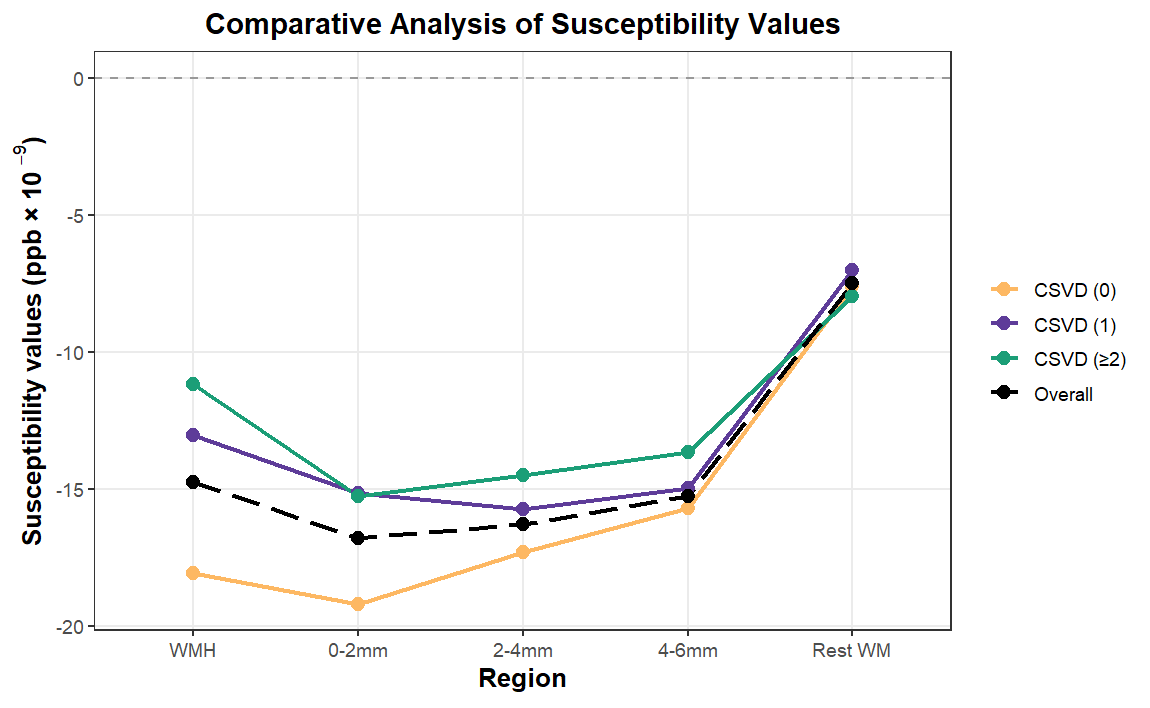


Mean quantitative susceptibility mapping (QSM) values in WMH cores, perilesional rims, and normal-appearing white matter, stratified by CSVD burden (0, 1, ≥2).

**Figure S5**


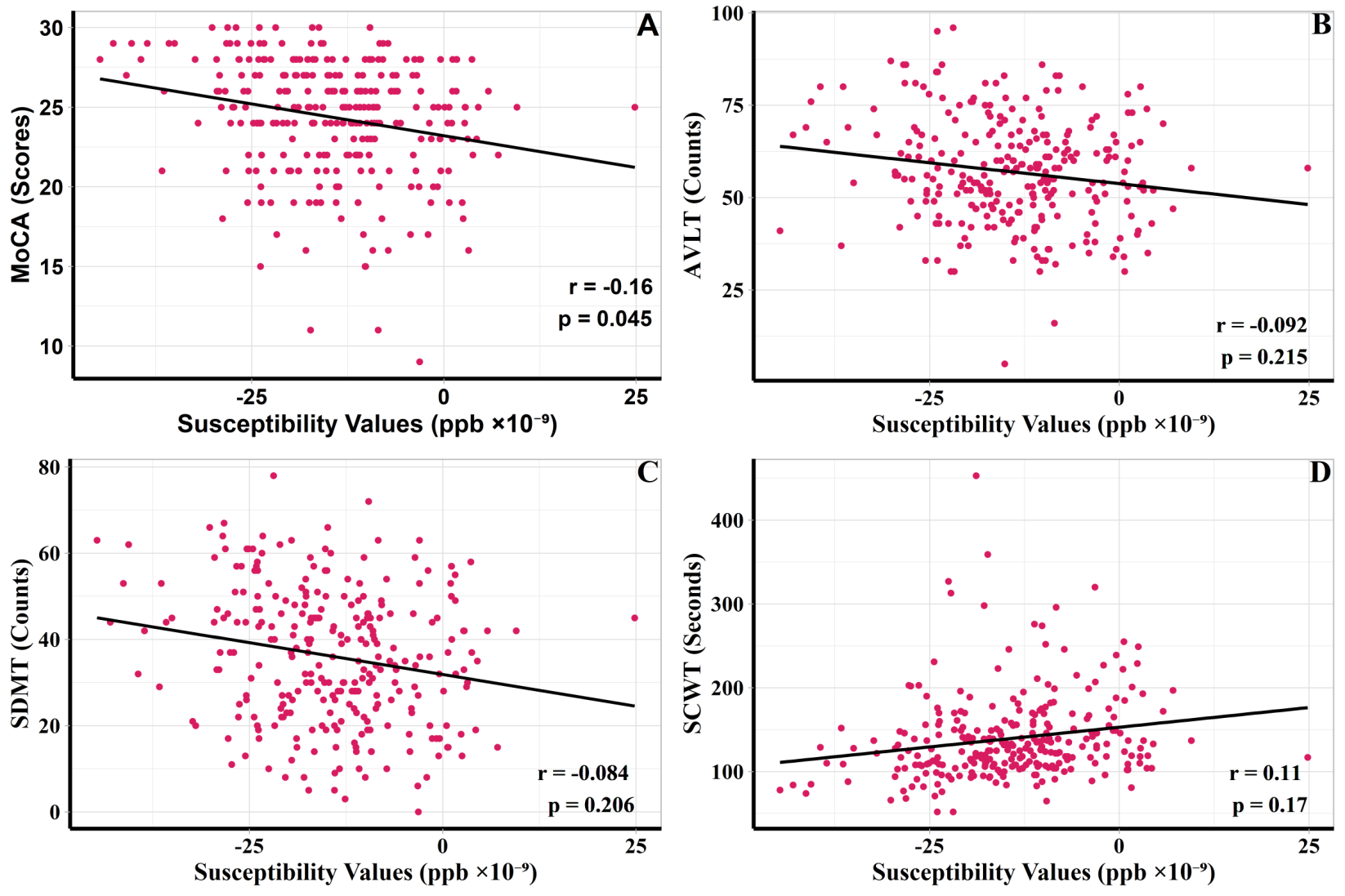


The line graph reflects the relationship between the susceptibility values of WMH and cognitive parameters. **(A, B, C, D)**. The first figure **(A)** shows a significant correlation between higher susceptibility values and lower MoCA scores. The r represents the partial correlation coefficient adjusted for the severity of CSVD and the ratio of the volume of WMH to total brain volume.

Abbreviations: MoCA = Montreal Cognitive Assessment, AVLT = Auditory Verbal Learning Test, SDMT = Symbol Digit Modalities Test, SCWT = Stroop Color and Word Test. ppb = parts per billion. WMH = white matter hyperintensities, CSVD = cerebral small vessel disease.

**Figure S6：DTI Metrics across CSVD Burden**


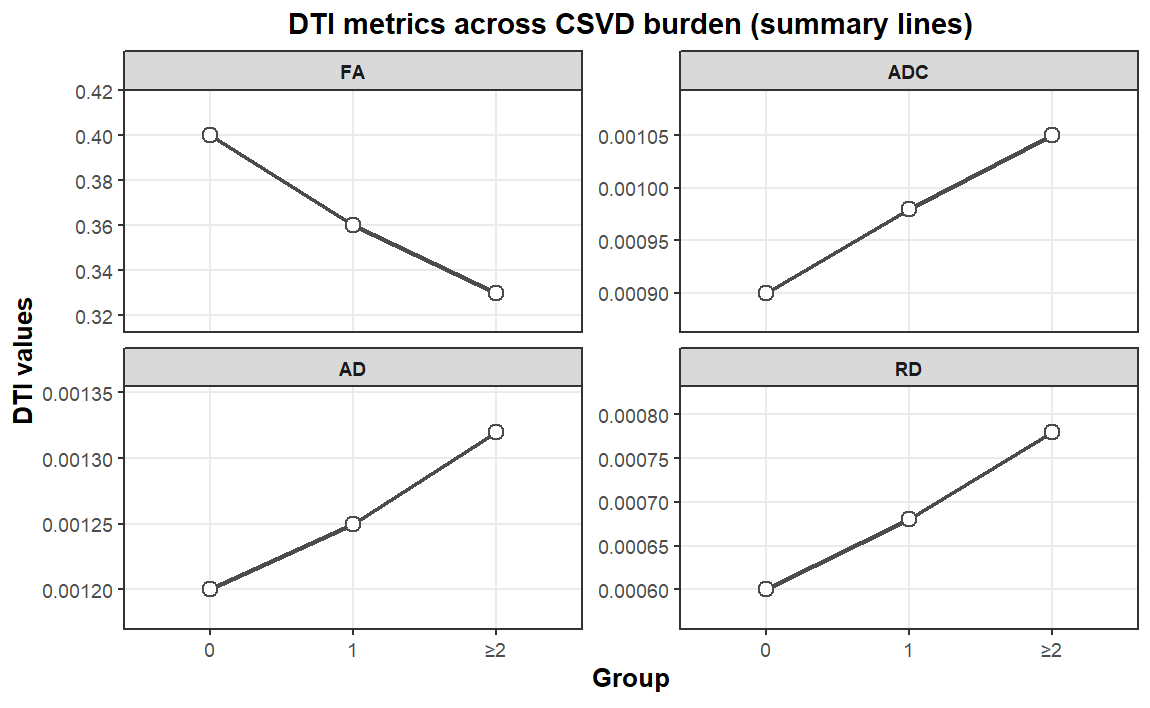


Mean DTI values stratified by CSVD burden. FA (fractional anisotropy) measures structural integrity; ADC (apparent diffusion coefficient), AD (axial diffusivity), and RD (radial diffusivity) measure water diffusion.

**Figure S7** The heatmap illustrates the P-values of WMH volume changes on cerebral white matter fiber tracts during the progression of CSVD


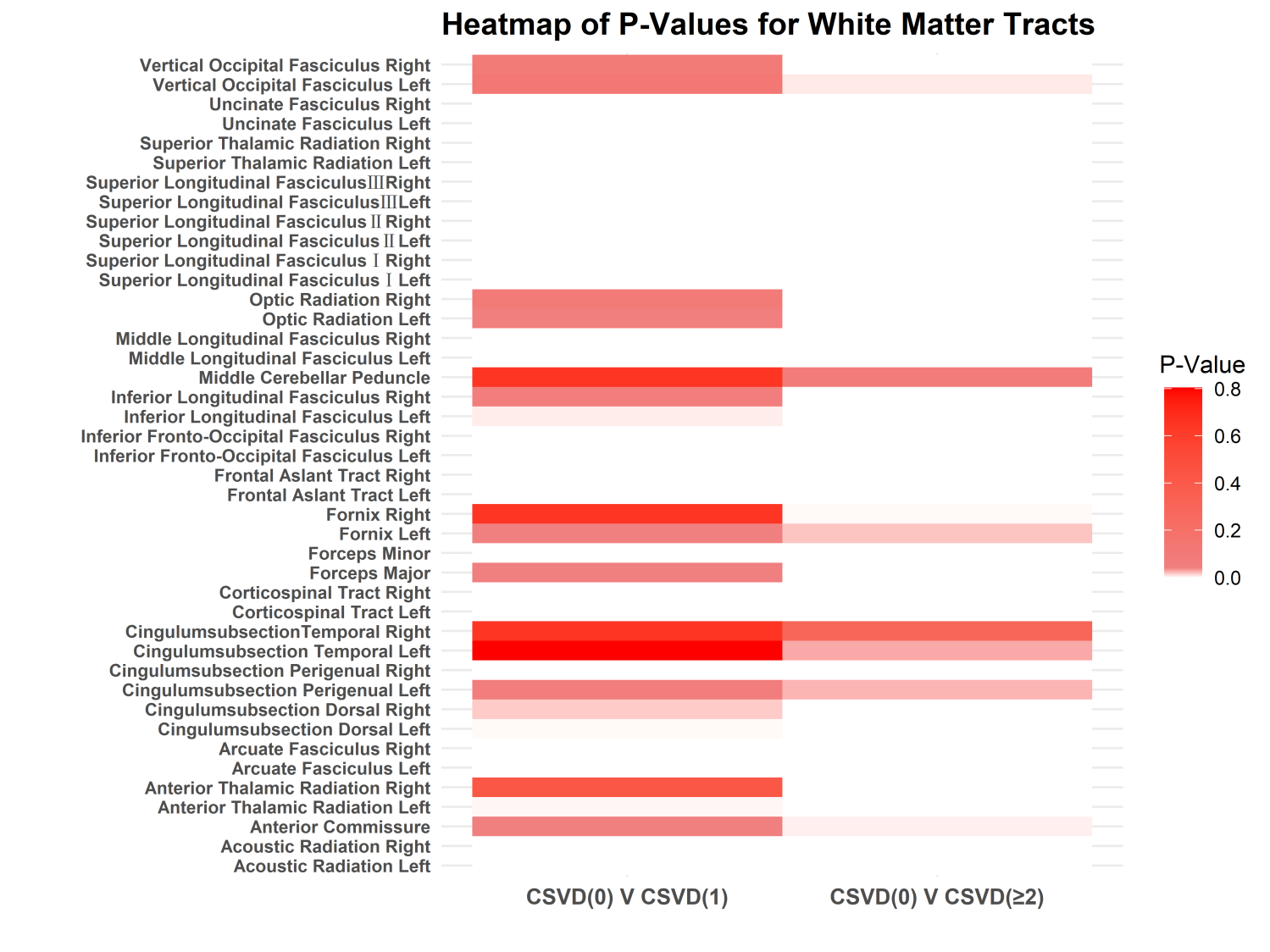


The left column shows the P-values for CSVD (1) compared to CSVD (0), while the right column represents the P-values for CSVD (≥2) compared to CSVD (0). The color spectrum, ranging from near-white (P < .05, statistically significant) to light coral and red (P ≥ .05, not statistically significant), indicates the magnitude of the P-values. This gradation in color highlights the significance of damage across various white matter fiber tracts. In the CSVD (1) group, fiber tracts such as Optic Radiation Left, Optic Radiation Right, Fornix Right, Anterior Thalamic Radiation Right, and Fornix Right show no substantial differences compared to CSVD (0). However, in the CSVD (≥2) group, these tracts display increasingly significant differences, suggesting minimal impact in the initial phase, which escalates in the CSVD (≥2) group.

Abbreviations: WMH = white matter hyperintensities. CSVD (0) = Score for burden of CSVD = 0, CSVD (1) = Score for burden of CSVD = 1, CSVD (≥2) = Score for burden of CSVD = 2,3,4, CSVD = cerebral small vessel disease.

**Table S1**  The abbreviation for white matter fiber tracts.

| **Tract** | **Abbreviation** |
| --- | --- |
| Arcuate Fasciculus | AF |
| Acoustic Radiation | AR |
| Anterior Thalamic Radiation | ATR |
| Cingulum subsection : Dorsal | CBD |
| Cingulum subsection : Peri-genual | CBP |
| Cingulum subsection : Temporal | CBT |
| Corticospinal Tract | CST |
| Frontal Aslant | FA |
| Forceps Major | FMA |
| Forceps Minor | FMI |
| Fornix | FX |
| Inferior Longitudinal Fasciculus | ILF |
| Inferior Fronto-Occipital Fasciculus | IFO |
| Middle Cerebellar Peduncle | MCP |
| Middle Longitudinal Fasciculuc | MdLF |
| Optic Radiation | OR |
| Superior Thalamic Radiation | STR |
| Superior Longitudinal Fasciculus 1 | SLF1 |
| Superior Longitudinal Fasciculus 2 | SLF2 |
| Superior Longitudinal Fasciculus 3 | SLF3 |
| Anterior Commissure | AC |
| Uncinate Fasciculus | UF |
| Vertical Occipital Fasciculus | VOF |

**Table S2 . Clinical Characteristics of the Participant.**

| **Variables** | **N** | **Descriptive values** |
| --- | --- | --- |
| QSM of WMH (ppb ×10^-9^) | 287 | -14.11（-20.89，-8.36） |
| QSM of NAWM (ppb ×10^-9^) | 287 | -7.7302（-13.04，-3.09） |
| Volume of WMH (mm3） | 287 | 751.46（310.08，2649.90） |
| TIV (cm^3^) | 294 | 1477.20±141.08 |
| Aβ1-42 | 191 | 161.42±35.11 |
| t-Tau | 191 | 142.30±42.41 |
| p-Tau181 | 191 | 291.98±63.91 |
| Male | 153/299 | 51.20% |
| Age (y) |  |  |
| ≤50 | 64/299 | 21.40% |
| ≤60 | 104/299 | 34.80% |
| ≤70 | 98/299 | 32.80% |
| ≥71 | 33/299 | 11.00% |
| Education (y) | |  |
| ≤6 | 75/296 | 25.30% |
| ≤12 | 75/296 | 25.30% |
| >12 | 146/296 | 49.40% |
| BMI |  |  |
| <24 | 118/293 | 40.30% |
| <28 | 123/293 | 42.00% |
| ≥28 | 52/293 | 17.70% |
| Hypertension | |  |
| no | 182/296 | 61.50% |
| yes | 114/296 | 38.50% |
| Diabetes |  |  |
| no | 168/297 | 56.60% |
| yes | 129/297 | 43.40% |
| Hyperlipidemia | |  |
| no | 169/288 | 58.70% |
| yes | 119/288 | 41.30% |
| Smoke |  |  |
| no | 230/299 | 76.90% |
| yes | 69/299 | 24.10% |
| Drink |  |  |
| no | 203/299 | 67.90% |
| yes | 96/299 | 32.10% |
| APOE ε4 |  |  |
| no | 263/299 | 88.00% |
| yes | 36/299 | 12.00% |
| CSVD Score | |  |
| Score0 | 171/299 | 57.20% |
| Score1 | 70/299 | 23.40% |
| Score2 | 32/299 | 10.70% |
| Score3 | 21/299 | 7.00% |
| Score4 | 5/299 | 1.70% |
| MoCA | 294 | 25(22,27) |
| AVLT | 294 | 56(48,67) |
| SDMT | 294 | 37(24,47) |
| SCWT | 294 | 127.5(107,155.25) |
| TSUM | 291 | 204(140,299) |

*Note*: Data are represented in percentages unless explicitly noted otherwise.

Abbreviations: QSM = quantitative susceptibility mapping，WMH = white matter hyperintensities, NAWM = normal appearing white matter, TIV = total intracranial volume, GM = gray matter, WM = white matter, APOE ε4 = Apolipoprotein E4, CSVD: cerebral small vessel disease, MoCA = Montreal Cognitive Assessment, AVLT = Auditory Verbal Learning Test, SDMT = Symbol Digit Modalities Test, SCWT = Stroop Color and Word Test, TSUM = TMT-A + TMT-B (TMT-A = Trail Making Test Part A, TMT-B = Trail Making Test Part B).

**Table S3.** **Demographic, Clinical, Imaging, and Cognitive Characteristics by CSVD Severity Groups**

| **Demographics** | **CSVD (0)** | **CSVD (1)** | **CSVD (≥2)** | ***p*** | **Statistical test** |
| --- | --- | --- | --- | --- | --- |
|  | **(n=171)** | **(n=70)** | **(n=58)** |  |  |
| Age, median [IQR] | 57 [48, 63] | 62 [56, 67] | 65.5 [60, 70] | <0.001 | H = 102.48 |
| Gender, n (%) |  | | | | |
| Male | 82 (48.0) | 33 (47.1) | 38 (65.5) | 0.012 | χ² = 5.94 |
| Female | 89 (52.0) | 37 (52.9) | 20 (34.5) |  |  |
| Education, median [IQR] | 14.01 [11.0, 16.0] | 12.00 [9.0, 15.0] | 11.21 [8.0, 14.0] | <0.001 | H = 35.67 |
| BMI, median [IQR] | 24.42 [22.1, 27.3] | 24.65 [22.8, 27.9] | 25.12 [23.2, 28.4] | 0.328 | H = 2.23 |
| Clinical characteristics, n (%) | | | | | |
| Hypertension |  | | | | |
| No | 119 (69.6) | 38 (54.3) | 28 (48.3) | 0.013 | χ² = 22.00 |
| Yes | 52 (30.4) | 32 (45.7) | 30 (51.7) |  |  |
| Diabetes |  | | | | |
| No | 113 (66.1) | 34 (48.6) | 23 (39.7) | 0.012 | χ² = 19.56 |
| Yes | 58 (33.9) | 36 (51.4) | 35 (60.3) |  |  |
| Hyperlipidemia |  | | | | |
| No | 106 (62.0) | 41 (58.6) | 33 (56.9) | 0.418 | χ² = 1.74 |
| Yes | 65 (38.0) | 29 (41.4) | 25 (43.1) |  |  |
| Smoke |  | | | | |
| No | 132 (77.2) | 55 (78.6) | 43 (74.1) | 0.632 | χ² = 0.92 |
| Yes | 39 (22.8) | 15 (21.4) | 15 (25.9) |  |  |
| Drink |  | | | | |
| No | 119 (69.6) | 46 (65.7) | 38 (65.5) | 0.34 | χ² = 2.16 |
| Yes | 52 (30.4) | 24 (34.3) | 20 (34.5) |  |  |
| Imaging and cognitive functions | | | | | |
| QSM of WMH, median [IQR] | -17.10 [-23.97, -10.05] | -12.34 [-18.91, -7.88] | -9.89 [-14.72, -2.07] | <0.001 | H = 45.82 |
| QSM of NAWM, median [IQR] | -8.12 [-13.54, -2.70] | -8.34 [-12.56, -4.12] | -6.59 [-11.52, -1.66] | 0.328 | H = 2.23 |
| MoCA, median [IQR] | 26.00 [23.00, 28.00] | 24.00 [21.00, 26.00] | 24.00 [21.00, 25.00] | 0.001 | H = 32.15 |
| AVLT, mean ± SD | 58.00 ± 13.28 | 56.00 ± 12.94 | 52.00 ± 13.42 | 0.009 | F = 8.83 |
| SDMT, mean ± SD | 44.00 ± 14.29 | 32.00 ± 13.47 | 26.00 ± 13.61 | <0.001 | F = 27.57 |
| SCWT, median [IQR] | 117.00 [102.00, 144.00] | 135.00 [112.00, 167.00] | 140.00 [126.00, 199.00] | <0.001 | H = 25.63 |

Abbreviations: QSM = quantitative susceptibility mapping，WMH = white matter hyperintensities, NAWM = normal appearing white matter, TIV = total intracranial volume, GM = gray matter, WM = white matter, CSVD: cerebral small vessel disease, MoCA = Montreal Cognitive Assessment, AVLT = Auditory Verbal Learning Test, SDMT = Symbol Digit Modalities Test, SCWT = Stroop Color and Word Test.

| **Table S4. Baseline demographics, cognitive performance, and plasma AD biomarkers across WMH burden groups** | | | | | | | |  |
| --- | --- | --- | --- | --- | --- | --- | --- | --- |
|  |  |  |  |  |  |  |  |  |
| **Variable** | **Overall (N=299)** | **WMH=0 (N=120)** | **WMH=1 (N=114)** | **WMH=2 (N=26)** | **WMH=3 (N=39)** | **P value** | **Statistical test** |  |
| Gender (Male %) | 51.20% | 42.50% | 51.80% | 65.40% | 66.70% | 0.024 | Chi-square, χ²=9.48 |  |
| Age (years) | 59.0 [52.0, 66.0] | 53.0 [43.8, 60.0] | 61.0 [55.0, 66.0] | 64.5 [56.0, 71.0] | 66.0 [61.0, 71.0] | <0.001 | Kruskal-Wallis, H=66.59 |  |
| MoCA | 25.0 [22.0, 27.0] | 26.0 [24.0, 28.0] | 24.0 [22.0, 26.0] | 24.0 [21.0, 26.0] | 24.0 [20.0, 25.0] | <0.001 | Kruskal-Wallis, H=30.37 |  |
| AVLT | 56.0 [48.0, 67.0] | 58.0 [51.0, 73.0] | 56.5 [48.0, 65.0] | 52.0 [42.0, 59.0] | 52.0 [42.0, 61.0] | <0.001 | Kruskal-Wallis, H=19.02 |  |
| SDMT | 37.0 [25.0, 47.0] | 45.0 [35.5, 56.0] | 32.5 [23.0, 43.3] | 29.0 [20.0, 42.0] | 26.0 [17.0, 33.0] | <0.001 | Kruskal-Wallis, H=55.92 |  |
| SCWT | 127.5 [107.0, 155.0] | 110.0 [95.0, 137.0] | 136.5 [111.5, 165.5] | 147.0 [118.0, 187.0] | 138.0 [124.0, 199.0] | <0.001 | Kruskal-Wallis, H=43.81 |  |
| Aβ1-42 (pg/mL) | 161.4 ± 35.1 | 161.1 ± 37.9 | 165.0 ± 30.7 | 148.3 ± 28.3 | 160.0 ± 39.4 | 0.474 | ANOVA, F=0.84 |  |
| Tau (pg/mL) | 142.3 ± 42.4 | 139.1 ± 42.0 | 146.6 ± 42.8 | 145.8 ± 44.1 | 140.3 ± 43.9 | 0.724 | ANOVA, F=0.44 |  |
| P-Tau181 (pg/mL) | 292.0 ± 63.9 | 283.4 ± 65.4 | 299.3 ± 62.0 | 275.4 ± 53.7 | 313.5 ± 64.2 | 0.113 | ANOVA, F=2.02 |  |
| **Note:** WMH = white matter hyperintensity; MoCA = Montreal Cognitive Assessment; AVLT = Auditory Verbal Learning Test; SDMT = Symbol Digit Modalities Test; SCWT = Stroop Color-Word Test. | | | | | | | |  |
|  |  |  |  |  |  |  |  |  |

| **Table S5. Associations between QSM-derived susceptibility and plasma AD biomarkers across WMH and perilesional regions** | | | | | |
| --- | --- | --- | --- | --- | --- |
|  |  |  |  |  |  |
| Plasma biomarker | Region | β | 95% CI | P value | FDR-adjusted P |
| **Aβ1-42** |  |  |  |  |  |
|  | 0–2 mm rim | 0.03 | −0.353 to 0.413 | 0.878 | 0.941 |
|  | 2–4 mm rim | 0.086 | −0.306 to 0.478 | 0.668 | 0.941 |
|  | 4–6 mm rim | 0.05 | −0.362 to 0.463 | 0.811 | 0.941 |
|  | >6 mm NAWM | −0.057 | −0.536 to 0.422 | 0.815 | 0.941 |
|  | WMH core | 0.013 | −0.354 to 0.379 | 0.946 | 0.946 |
| **Phosphorylated tau-181** |  |  |  |  |  |
|  | 0–2 mm rim | −0.306 | −0.891 to 0.279 | 0.305 | 0.717 |
|  | 2–4 mm rim | −0.147 | −0.706 to 0.411 | 0.605 | 0.941 |
|  | 4–6 mm rim | −0.096 | −0.665 to 0.473 | 0.741 | 0.941 |
|  | >6 mm NAWM | 0.056 | −0.569 to 0.682 | 0.86 | 0.941 |
|  | WMH core | −0.269 | −0.817 to 0.278 | 0.335 | 0.717 |
| **Total tau** |  |  |  |  |  |
|  | 0–2 mm rim | 0.443 | 0.131 to 0.755 | 0.005 | **0.041** |
|  | 2–4 mm rim | 0.397 | 0.088 to 0.705 | 0.012 | 0.059 |
|  | 4–6 mm rim | 0.362 | 0.060 to 0.665 | 0.019 | 0.071 |
|  | >6 mm NAWM | 0.265 | −0.039 to 0.569 | 0.087 | 0.262 |
|  | WMH core | 0.52 | 0.191 to 0.850 | 0.002 | **0.029** |
| Linear regression models adjusted for age, sex, and education. CI = confidence interval; FDR = false discovery rate; QSM = quantitative susceptibility mapping; WMH = white matter hyperintensity; NAWM = normal-appearing white matter. Bold values indicate statistical significance after FDR correction (P < 0.05). | | | | | |
|  |  |  |  |  |  |

| **Table . Associations between WMH volume and plasma AD biomarkers** | | | | |
| --- | --- | --- | --- | --- |
|  |  |  |  |  |
| Plasma biomarker | β (×10⁻³) | 95% CI (×10⁻³) | P value | FDR-adjusted P |
| Aβ1-42 | −1.38 | −2.89 to 0.13 | 0.074 | 0.268 |
| Total tau | −0.76 | −1.75 to 0.23 | 0.134 | 0.268 |
| Phosphorylated tau-181 | −0.33 | −2.26 to 1.60 | 0.735 | 0.837 |
| Linear regression models adjusted for age, sex, and education. CI = confidence interval; FDR = false discovery rate; WMH = white matter hyperintensity. | | | | |
|  |  |  |  |  |

|  | **Table S6:** Comparative Analysis of Susceptibility Values of WMH | | | | | | | |  |
| --- | --- | --- | --- | --- | --- | --- | --- | --- | --- |
|  | Test Variable | CSVD (0) | CSVD (1) | CSVD (≥2) | *P* | Pair-Wise Test | | |  |
|  |  |  |  |  |  | a | b | c |  |
|  | A | -17.10（-23.97, -10.05） | -12.34（-18.91, -7.88） | -9.89 (-14.72, -2.07) | <.001 | .004 | <.001 | .02 |  |
|  | Note. —Pair-wise test (Tukey’s test): a = CSVD (1) vs. CSVD (0), b = CSVD (≥2) vs. CSVD (0), c = CSVD (1) vs. CSVD (≥2). CSVD (0) = Score for burden of CSVD = 0, CSVD (1) = Score for burden of CSVD = 1, CSVD (≥2) = Score for burden of CSVD = 2,3,4, CSVD = cerebral small vessel disease. A = susceptibility values of WMH. WMH = white matter hyperintensities.  P-values derived from multiple comparisons have undergone False Discovery Rate (FDR) correction.  **Table S7. Comparison of QSM Values Among Different Regions in Total Population (N=246)**   \| **WMH** \| **0-2mm** \| **2-4mm** \| **4-6mm** \| **P** \| \| --- \| --- \| --- \| --- \| --- \| \| -14.74 (-22.49, -8.71) \| -16.78 (-23.29, -10.51) \| -16.28 (-22.69, -9.54) \| -15.25 (-20.70, -8.40) \| <.001 \|   Data are expressed as median (interquartile range). P value from Friedman test.  Abbreviations: WMH = white matter hyperintensities; 0-2mm = 0-2mm region surrounding WMH; 2-4mm = 2-4mm region surrounding WMH; 4-6mm = 4-6mm region surrounding WMH.  **Table S8. Pairwise Comparisons of QSM Values Between Different Regions in Overall Population**   \| **Region Comparison** \| **Region 1** \| **Region 2** \| **Median Difference** \| **P-value*** \| \| --- \| --- \| --- \| --- \| --- \| \| WMH vs 0-2mm \| -14.74 (-22.49, -8.71) \| -16.78 (-23.29, -10.51) \| 1.26 (-1.26, 3.81) \| <0.001 \| \| WMH vs 2-4mm \| -14.74 (-22.49, -8.71) \| -16.28 (-22.69, -9.54) \| 0.31 (-2.26, 3.74) \| <0.001 \| \| WMH vs 4-6mm \| -14.74 (-22.49, -8.71) \| -15.25 (-20.70, -8.40) \| -0.75 (-4.19, 3.00) \| <0.001 \| \| 0-2mm vs 2-4mm \| -16.78 (-23.29, -10.51) \| -16.28 (-22.69, -9.54) \| -0.47 (-2.16, 0.78) \| 0.002 \| \| 0-2mm vs 4-6mm \| -16.78 (-23.29, -10.51) \| -15.25 (-20.70, -8.40) \| -1.51 (-4.53, 0.19) \| <0.001 \| \| 2-4mm vs 4-6mm \| -16.28 (-22.69, -9.54) \| -15.25 (-20.70, -8.40) \| -1.01 (-2.62, -0.20) \| <0.001 \|   Note. —Data are expressed as median (interquartile range). P values are from Wilcoxon signed-rank test for paired comparisons, and P values were false discovery rate (FDR) corrected. Abbreviations: WMH = white matter hyperintensities; 0-2mm = 0-2mm region surrounding WMH; 2-4mm = 2-4mm region surrounding WMH; 4-6mm = 4-6mm region surrounding WMH.  **Table S9. Extended Spatial Analysis of Susceptibility Values in Regions Beyond 6mm from WMH**   \| Test Variable \| CSVD (0) \| CSVD (1) \| CSVD (≥2) \| P \| \| --- \| --- \| --- \| --- \| --- \| \| 6-8mm \| -13.82 (-19.45, -7.86) \| -13.21 (-18.74, -7.52) \| -12.95 (-18.83, -7.18) \| 0.512 \| \| 8-10mm \| -11.26 (-16.78, -5.94) \| -10.83 (-16.12, -5.61) \| -11.04 (-16.95, -5.73) \| 0.623 \| \| Rest of WM (>10mm) \| -6.85 (-11.94, -2.68) \| -6.42 (-11.53, -2.31) \| -7.08 (-12.47, -2.89) \| 0.687 \|  \| **Table S10：** Exploring the Influence of Varied Demographic Profiles on Susceptibility Values of WMH \| \| \| \| \| \| \| --- \| --- \| --- \| --- \| --- \| --- \| \|  \| **Variables** \| **Susceptibility Values of WMH** \| ***Z*** \| ***P* Value** \|  \| \|  \| Gender \|  \| 0.78 \| .38 \|  \| \|  \| Male \| -13.68 (-19.63, -8.28) \| \| \|  \| \|  \| Female \| -15.42 (-22.32, -8.53) \| \| \|  \| \|  \| Age (y) \|  \| 18.66 \| <.001 \|  \| \|  \| ≤50 \| -22.20 (-27.80, -11.74) \| \| \|  \| \|  \| ≤60 \| -14.21 (-18.91, -7.03) \| \| \|  \| \|  \| ≤70 \| -12.20 (-19.61, -8.65) \| \| \|  \| \|  \| ≥71 \| -11.27 (-17.94, -4.87) \| \| \|  \| \|  \| Education (y) \|  \| 6.14 \| .046 \|  \| \|  \| ≤6 \| -13.54 (-20.36, -9.21) \| \| \|  \| \|  \| ≤12 \| -11.72 (-17.13, -6.66) \| \| \|  \| \|  \| >12 \| -15.99 (-23.71, -8.50) \| \| \|  \| \|  \| BMI \|  \| 1.53 \| .465 \|  \| \|  \| <24 \| -13.81 (-22.51, -7.12) \| \| \|  \| \|  \| <28 \| -14.23 (-20.89, -9.85) \| \| \|  \| \|  \| ≥28 \| -13.98 (-18.91, -7.24) \| \| \|  \| \|  \| Hypertension \| \| 9.11 \| .003 \|  \| \|  \| No \| -15.99 (-23.43, -9.35) \| \| \|  \| \|  \| Yes \| -12.63 (-17.04, -6.86) \| \| \|  \| \|  \| Diabetes \|  \| 13.17 \| <.001 \|  \| \|  \| No \| -16.65 (-23.57, -9.67) \| \| \|  \| \|  \| Yes \| -11.19 (-17.59, -6.11) \| \| \|  \| \|  \| Hyperlipidemia \| \| 0.77 \| .38 \|  \| \|  \| No \| -14.05 (-23.57, -8.46) \| \| \|  \| \|  \| Yes \| -14.21 (-18.44, -8.51) \| \| \|  \| \|  \| Smoke \|  \| 0.08 \| .77 \|  \| \|  \| No \| -14.213 (-21.340, -8.455) \| \| \|  \| \|  \| Yes \| -13.543 (-19.957, -8.681) \| \| \|  \| \|  \| Drink \|  \| 0.002 \| .96 \|  \| \|  \| No \| -14.22 (-21.06, -8.41) \| \| \|  \| \|  \| Yes \| -13.88 (-20.89, -9.00) \| \| \|  \| \|  \| APOE4 \|  \| 3.39 \| .07 \|  \| \|  \| No \| -13.36 (-18.90, -7.26) \| \| \|  \| \|  \| Yes \| -16.43 (-24.61, -9.96) \| \| \|  \| \|  \| CSVD \|  \| 28.78 \| <.001 \|  \| \|  \| CSVD (0) \| -17.10 (-23.97, -10.05) \| \| \|  \| \|  \| CSVD (1) \| -12.34 (-18.91, -7.88) \| \| \|  \| \|  \| CSVD (≥2) \| - 9.89 (-14.73, -2.07) \| \| \|  \| \|  \| Note. —WMH = white matter hyperintensities, CSVD (0) = Score for burden of CSVD = 0, CSVD (1) = Score for burden of CSVD = 1, CSVD (≥2) = Score for burden of CSVD = 2,3,4. CSVD = cerebral small vessel disease. APOE4 = Apolipoprotein E4.  Data are medians, with IQRs in parentheses. \| \| \| \|  \| | | | | | | | |  |

|  | **Table S11：** Exploring the Relationship between Susceptibility to WMH and Key Prescreening Variables   \| **Predictor** \| **B** \| **Standard Error, SE** \| **Wald** \| ***P*** \| **Odds ratio** \| \| --- \| --- \| --- \| --- \| --- \| --- \| \| Hypertension \| \|  \|  \|  \|  \| \| No \| -0.27 \| 0.28 \| 0.92 \| 0.34 \| 0.76 \| \| Yes \| Reference \| —— \| —— \| —— \| —— \| \| Diabetes \|  \|  \|  \|  \|  \| \| No \| -0.53 \| 0.27 \| 3.94 \| 0.047 \| 0.59 \| \| Yes \| Reference \| —— \| —— \| —— \| —— \| \| CSVD \|  \|  \|  \|  \|  \| \| CSVD (0) \| -1.33 \| 0.41 \| 10.66 \| 0.001 \| 0.27 \| \| CSVD (1) \| -0.68 \| 0.41 \| 2.76 \| 0.1 \| 0.51 \| \| CSVD (≥2) \| Reference \| —— \| —— \| —— \| —— \| \| Age (y) \|  \|  \|  \|  \|  \| \| ≤50 \| -0.34 \| 0.55 \| 0.39 \| 0.54 \| 0.71 \| \| ≤60 \| 0.13 \| 0.46 \| 0.08 \| 0.77 \| 1.14 \| \| ≤70 \| 0.43 \| 0.44 \| 0.95 \| 0.33 \| 1.54 \| \| ≥71 \| Reference \| —— \| —— \| —— \| —— \| \| Education (y) \| \|  \|  \|  \|  \| \| ≤6 \| 0.02 \| 0.34 \| 0.003 \| 0.96 \| 1.02 \| \| ≤12 \| 0.12 \| 0.34 \| 0.1 \| 0.75 \| 1.11 \| \| >12 \| Reference \| —— \| —— \| —— \| —— \|   *Note*: Abbreviations: CSVD (0) = Score for burden of CSVD = 0, CSVD (1) = Score for burden of CSVD = 1, CSVD (≥2) = Score for burden of CSVD = 2,3,4, CSVD = cerebral small vessel disease. WMH = white matter hyperintensities. |  |
| --- | --- | --- | --- | --- | --- | --- | --- | --- | --- | --- | --- | --- | --- | --- | --- | --- | --- | --- | --- | --- | --- | --- | --- | --- | --- | --- | --- | --- | --- | --- | --- | --- | --- | --- | --- | --- | --- | --- | --- | --- | --- | --- | --- | --- | --- | --- | --- | --- | --- | --- | --- | --- | --- | --- | --- | --- | --- | --- | --- | --- | --- | --- | --- | --- | --- | --- | --- | --- | --- | --- | --- | --- | --- | --- | --- | --- | --- | --- | --- | --- | --- | --- | --- | --- | --- | --- | --- | --- | --- | --- | --- | --- | --- | --- | --- | --- | --- | --- | --- | --- | --- | --- | --- | --- | --- | --- | --- | --- | --- | --- | --- | --- | --- | --- | --- | --- | --- | --- | --- | --- | --- | --- |
|  |  |  |
|  |  |  |
|  |  |  |
|  |  |  |
|  |  |  |
|  |  |  |
|  |  |  |
|  |  |  |
|  |  |  |

|  | **Table S12：** Partial Correlation: Associations between Susceptibility Values of WMH and Cognitive Tests | | | | | |
| --- | --- | --- | --- | --- | --- | --- |
|  |  |  |  |  |  |  |
|  |  |  |  |  |  |  |
|  | **Statistical Value** | **MoCA** | **AVLT** | **SDMT** | **SCWT** | **TSUM** |
|  |  |  |  |  |  |  |
|  |  |  |  |  |  |  |
|  | *r* | -0.16 | -0.09 | -0.08 | 0.11 | 0.07 |
|  | *p* | .009 | .13 | .17 | .07 | .25 |
|  | Adjusted*-p-*value | .045 | .22 | .21 | .17 | .25 |
|  | MoCA = Montreal Cognitive Assessment, AVLT = Auditory Verbal Learning Test, SDMT = Symbol Digit Modalities Test, SCWT = Stroop Color and Word Test, TSUM = TMT-A + TMT-B (TMT-A = Trail Making Test Part A, TMT-B = Trail Making Test Part B). WMH = white matter hyperintensities.  All P-values have undergone False Discovery Rate (FDR) adjustment (Adjusted-p-value). | | | | | |

**Table S13. Correlation Analysis between Regional QSM Values and Cognitive Function**

| **Test Variable** | **MoCA** | **AVLT** | **SDMT** | **SCWT** | **MUST** |
| --- | --- | --- | --- | --- | --- |
| **0-2mm QSM** | -0.160 (0.042/0.122) | -0.133 (0.114/0.191) | -0.175 (0.022/0.122) | 0.181 (0.018/0.122) | 0.180 (0.018/0.122) |
| **2-4mm QSM** | -0.126 (0.144/0.206) | -0.138 (0.095/0.186) | -0.157 (0.048/0.122) | 0.166 (0.033/0.122) | 0.163 (0.038/0.122) |
| **4-6mm QSM** | -0.111 (0.219/0.285) | -0.127 (0.137/0.206) | -0.136 (0.102/0.186) | 0.156 (0.049/0.122) | 0.153 (0.055/0.122) |
| **The rest of WM** | -0.110 (0.228/0.285) | -0.104 (0.264/0.310) | -0.042 (0.807/0.849) | 0.004 (0.998/0.998) | 0.060 (0.641/0.713) |

Data are presented as Spearman correlation coefficient (uncorrected P value/FDR-corrected P value).

Abbreviations: QSM, Quantitative Susceptibility Mapping; MoCA, Montreal Cognitive Assessment; AVLT, Auditory Verbal Learning Test; SDMT, Symbol Digit Modalities Test; SCWT, Stroop Color and Word Test; TSUM, Trail Making Test sum score; WM, white matter; FDR, False Discovery Rate.

|  | **Table S14:** Comparative Analysis of DTI Parameters | | | | | | | |  |  |
| --- | --- | --- | --- | --- | --- | --- | --- | --- | --- | --- |
|  | DTI | CSVD (1) （n=60） | CSVD (≥2) （n=54） | CSVD (0) （n=141） | P value (ANOVA) | Pair-Wise Test | | |  |  |
|  |  |  |  |  |  |  |  |  |  |  |
|  |  |  |  |  |  | a | b | c |  |  |
|  | ADC | 0.001(0.0001) | 0.001(0.0001) | 0.001(0.0001) | <.001 | <.001 | <.001 | .03 |  |  |
|  | FA | 0.36(0.63) 0.33(0.05) | | 0.40(0.06) | <.001 | <.001 | 0.001 | <.001 |  |  |
|  | RD | 0.001(0.0001) | .001(0.0001) | <0.001 | <.001 | <.001 | <.001 | .002 |  |  |
|  | AD | 0.001(0.0001) | .001(0.0001) | <0.001 | <.001 | <.001 | <.001 | .87 |  |  |
|  | Note. —Data are means (SD), Pair-wise test (Tukey’s test): a = CSVD (≥2) vs. CSVD (0), b = CSVD (≥2) vs. CSVD (1), c = CSVD (1) vs. CSVD (0). CSVD (0) = Score for burden of CSVD = 0, CSVD (1) = Score for burden of CSVD = 1, CSVD (≥2) = Score for burden of CSVD = 2,3,4, CSVD = cerebral small vessel disease, DTI: diffusion tensor imaging, FA: fractional anisotropy, RD: radial diffusivity, ADC: apparent diffusion coefficient, AD: axial diffusivity.  P-values derived from multiple comparisons have undergone False Discovery Rate (FDR) correction.   \|  \| **Table S15:** Comparative Analysis of White Matter Tracts \| \| \| \| \| \| \|  \| \| --- \| --- \| --- \| --- \| --- \| --- \| --- \| --- \| --- \| \|  \|  \| \|  \|  \| CSVD (0) VS. CSVD (1) \| \| \| CSVD (0) VS. CSVD (≥2) \| \| \|  \| \|  \|  \|  \| \|  \| White matter tract \| Z \| *P* \| Adjusted-P \| Z \| *P* \| Adjusted-*P* \|  \| \|  \| Anterior Commissure \| -2.11 \| .04 \| .049 \| -2.86 \| .004 \| .01 \|  \| \|  \| Arcuate Fasciculus Left \| -5.55 \| <.001 \| <.001 \| -8.78 \| <.001 \| <.001 \|  \| \|  \| Arcuate Fasciculus Right \| -5.17 \| <.001 \| <.001 \| -8.92 \| <.001 \| <.001 \|  \| \|  \| Anterior Thalamic Radiation Left \| -3.08 \| .002 \| .003 \| -5.66 \| <.001 \| <.001 \|  \| \|  \| Anterior Thalamic Radiation Right \| -0.88 \| .38 \| .42 \| -4.27 \| <.001 \| <.001 \|  \| \|  \| Acoustic Radiation Left \| -6.25 \| <.001 \| <.001 \| -9.64 \| <.001 \| <.001 \|  \| \|  \| Acoustic Radiation Right \| -6.13 \| <.001 \| <.001 \| -9.69 \| <.001 \| <.001 \|  \| \|  \| Cingulumsubsection Dorsal Left \| -3.38 \| .001 \| .002 \| -5.79 \| <.001 \| <.001 \|  \| \|  \| Cingulumsubsection Dorsal Right \| -2.54 \| .011 \| .02 \| -6.38 \| <.001 \| <.001 \|  \| \|  \| Cingulumsubsection Perigenual Left \| -1.95 \| .051 \| .07 \| -2.29 \| .02 \| .02 \|  \| \|  \| Cingulumsubsection Perigenual Right \| -3.77 \| <.001 \| <.001 \| -5.75 \| <.001 \| <.001 \|  \| \|  \| Cingulumsubsection Temporal Left \| -0.25 \| .80 \| .80 \| -2.2 \| .03 \| .03 \|  \| \|  \| CingulumsubsectionTemporal Right \| -0.49 \| .63 \| .65 \| -1.04 \| .30 \| .30 \|  \| \|  \| Corticospinal Tract Left \| -4.67 \| <.001 \| <.001 \| -9.77 \| <.001 \| <.001 \|  \| \|  \| Corticospinal Tract Right \| -3.74 \| <.001 \| <.001 \| -10.02 \| <.001 \| <.001 \|  \| \|  \| Frontal Aslant Tract Left \| -5.24 \| <.001 \| <.001 \| -8.89 \| <.001 \| <.001 \|  \| \|  \| Frontal Aslant Tract Right \| -5.44 \| <.001 \| <.001 \| -9.78 \| <.001 \| <.001 \|  \| \|  \| Forceps Major \| -2.08 \| .04 \| .05 \| -6.17 \| <.001 \| <.001 \|  \| \|  \| Forceps Minor \| -5.56 \| <.001 \| <.001 \| -8.86 \| <.001 \| <.001 \|  \| \|  \| Fornix Left \| -2.2 \| .03 \| .04 \| -2.38 \| .02 \| .02 \|  \| \|  \| Fornix Right \| -0.48 \| .64 \| .65 \| -3.03 \| .002 \| .002 \|  \| \|  \| Inferior Fronto-Occipital Fasciculus Left \| -5.92 \| <.001 \| <.001 \| -9.17 \| <.001 \| <.001 \|  \| \|  \| Inferior Fronto-Occipital Fasciculus Right \| -4.05 \| <.001 \| <.001 \| -8.84 \| <.001 \| <.001 \|  \| \|  \| Inferior Longitudinal Fasciculus Left \| -2.86 \| .004 \| .01 \| -5.29 \| <.001 \| <.001 \|  \| \|  \| Inferior Longitudinal Fasciculus Right \| -1.93 \| .053 \| .07 \| -6.14 \| <.001 \| <.001 \|  \| \|  \| Middle Cerebellar Peduncle \| -0.51 \| .61 \| .65 \| -1.72 \| 0.09 \| 0.09 \|  \| \|  \| Middle Longitudinal Fasciculus Left \| -4.34 \| <.001 \| <.001 \| -9.59 \| <.001 \| <.001 \|  \| \|  \| Middle Longitudinal Fasciculus Right \| -5.24 \| <.001 \| <.001 \| -9.43 \| <.001 \| <.001 \|  \| \|  \| Optic Radiation Left \| -2.05 \| .04 \| .054 \| -6.8 \| <.001 \| <.001 \|  \| \|  \| Optic Radiation Right \| -1.73 \| 0.08 \| 0.1 \| -6.96 \| <.001 \| <.001 \|  \| \|  \| Superior Longitudinal Fasciculus Ⅰ Left \| -5.78 \| <.001 \| <.001 \| -8.94 \| <.001 \| <.001 \|  \| \|  \| Superior Longitudinal Fasciculus Ⅰ Right \| -5.01 \| <.001 \| <.001 \| -8.84 \| <.001 \| <.001 \|  \| \|  \| Superior Longitudinal Fasciculus Ⅱ Left \| -5.46 \| <.001 \| <.001 \| -9.3 \| <.001 \| <.001 \|  \| \|  \| Superior Longitudinal Fasciculus Ⅱ Right \| -5.93 \| <.001 \| <.001 \| -8.81 \| <.001 \| <.001 \|  \| \|  \| Superior Longitudinal Fasciculus Ⅲ Left \| -4.19 \| <.001 \| <.001 \| -7.75 \| <.001 \| <.001 \|  \| \|  \| Superior Longitudinal Fasciculus Ⅲ Right \| -5.00 \| <.001 \| <.001 \| -8.1 \| <.001 \| <.001 \|  \| \|  \| Superior Thalamic Radiation Left \| -5.00 \| <.001 \| <.001 \| -10.4 \| <.001 \| <.001 \|  \| \|  \| Superior Thalamic Radiation Right \| -5.15 \| <.001 \| <.001 \| -9.91 \| <.001 \| <.001 \|  \| \|  \| Uncinate Fasciculus Left \| -5.495 \| <.001 \| <.001 \| -8.234 \| <.001 \| <.001 \|  \| \|  \| Uncinate Fasciculus Right \| -4.323 \| <.001 \| <.001 \| -8.152 \| <.001 \| <.001 \|  \| \|  \| Vertical Occipital Fasciculus Left \| -1.564 \| .12 \| .13 \| -2.769 \| .01 \| .01 \|  \| \|  \| Vertical Occipital Fasciculus Right \| -1.834 \| .07 \| .10 \| -4.743 \| <.001 \| <.001 \|  \| \| Note. —CSVD (0) = Score for burden of CSVD = 0, CSVD (1) = Score for burden of CSVD = 1, CSVD (≥2) = Score for burden of CSVD = 2,3,4, CSVD = cerebral small vessel disease. P-values derived from multiple comparisons have undergone False Discovery Rate (FDR) correction. \| \| \| \| \| \| \| \|  \| | | | | | | | | |  |
